# Supplementary material for: Identifying Personalized Metabolic Signatures in Breast Cancer
Source: Metabolites. 2020 Dec 30;11(1):20. doi: 10.3390/metabo11010020 (PMC7823382; doi:10.3390/metabo11010020)
Supplement: Supplementary file 1 [file metabolites-11-00020-s001.zip › Supplementary_files_revision/Supplementary_file_1_final.pdf]

Divergence Analysis

Contents

1 Introduction **1**

2 PCA with metabolic reaction data **1**

3 PCA with divergence data **1**

4 Examining clusters with important phenotypes **4**
4.1 PAM50 . . . 4
4.2 ER Status . . . 7
4.3 Triple Negative Status . . . 8
4.4 AJCC Stage . . . 9
4.5 Percent lymphocyte infiltration . . . 10
4.6 Percent monocyte infiltration . . . 10
4.7 Percent necrosis . . . 10
4.8 Percent neutrophil infiltration . . . 11
4.9 Percent normal cells . . . 11
4.10 Percent stromal cells . . . 11
4.11 Percent tumor cells . . . 12
4.12 Tissue source site . . . 12
4.13 Year of initial pathologic diagnosis . . . 13
4.14 Pathologic state . . . 14

5 Reaction value analysis **15**

6 Analysis of divergence data **19**

7 Molecular Checks **22**

1 Introduction

2 PCA with metabolic reaction data

On each plot we color the samples according to the 4 clusters that have been identified through k-means.

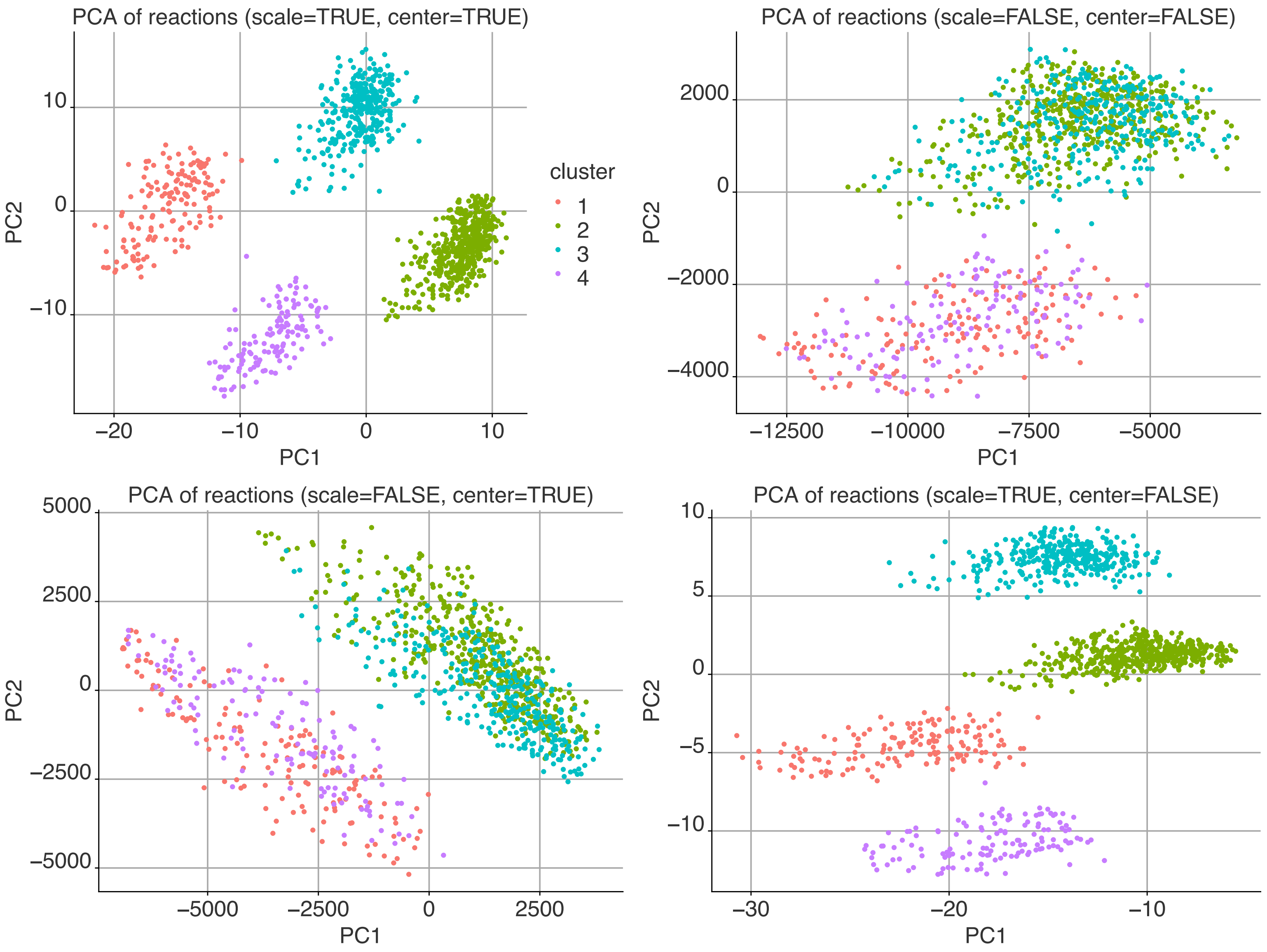

3 PCA with divergence data

We cluster the divergence data and color the plots with the same cluster scheme as above.

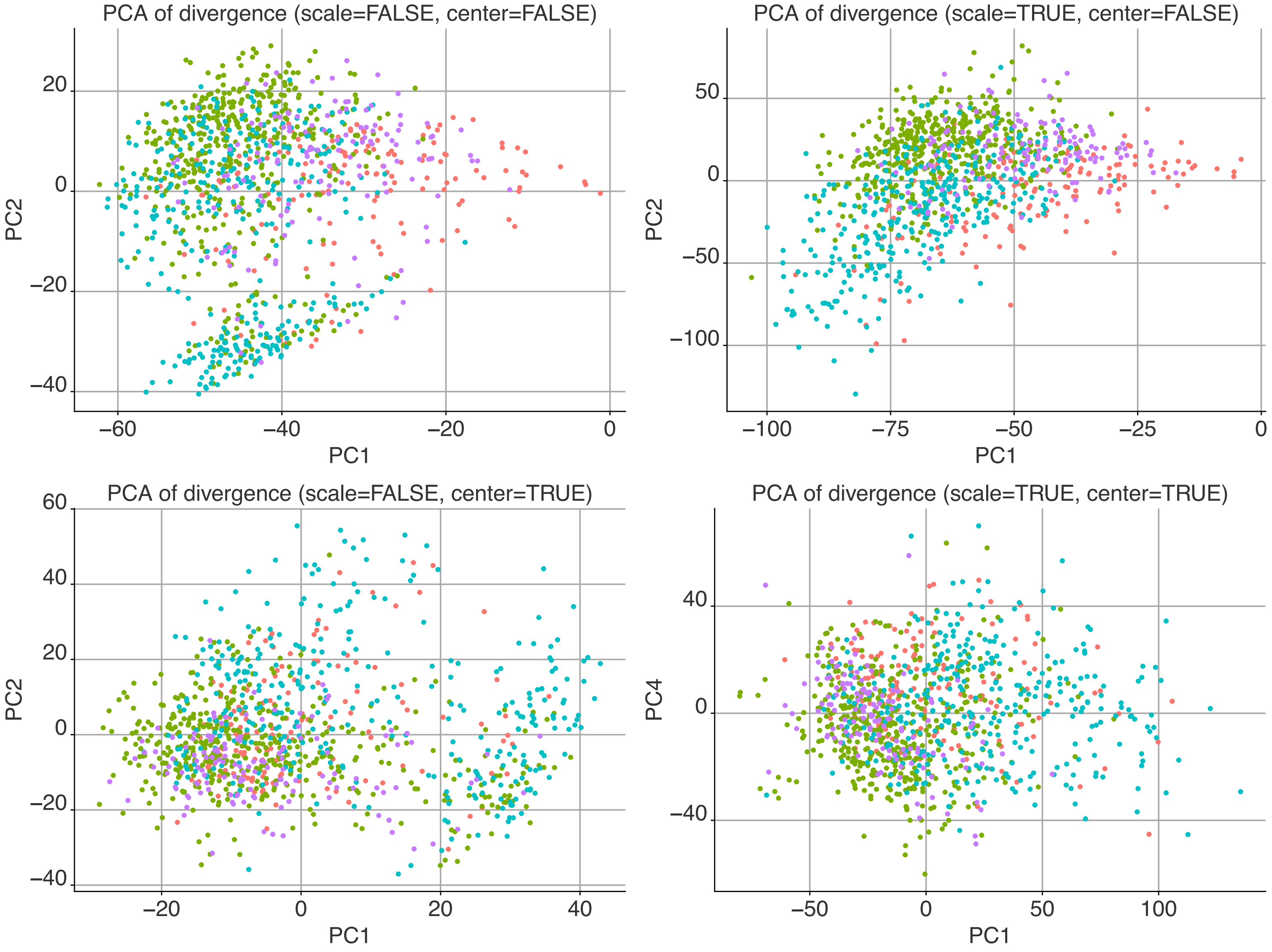
PCA of divergence (scale=FALSE, center=FALSE)
PC1
PC2
PCA of divergence (scale=TRUE, center=FALSE)
PC1
PC2
PCA of divergence (scale=FALSE, center=TRUE)
PC1
PC2
PCA of divergence (scale=TRUE, center=TRUE)
PC1
PC4

4 Examining clusters with important phenotypes

4.1 PAM50

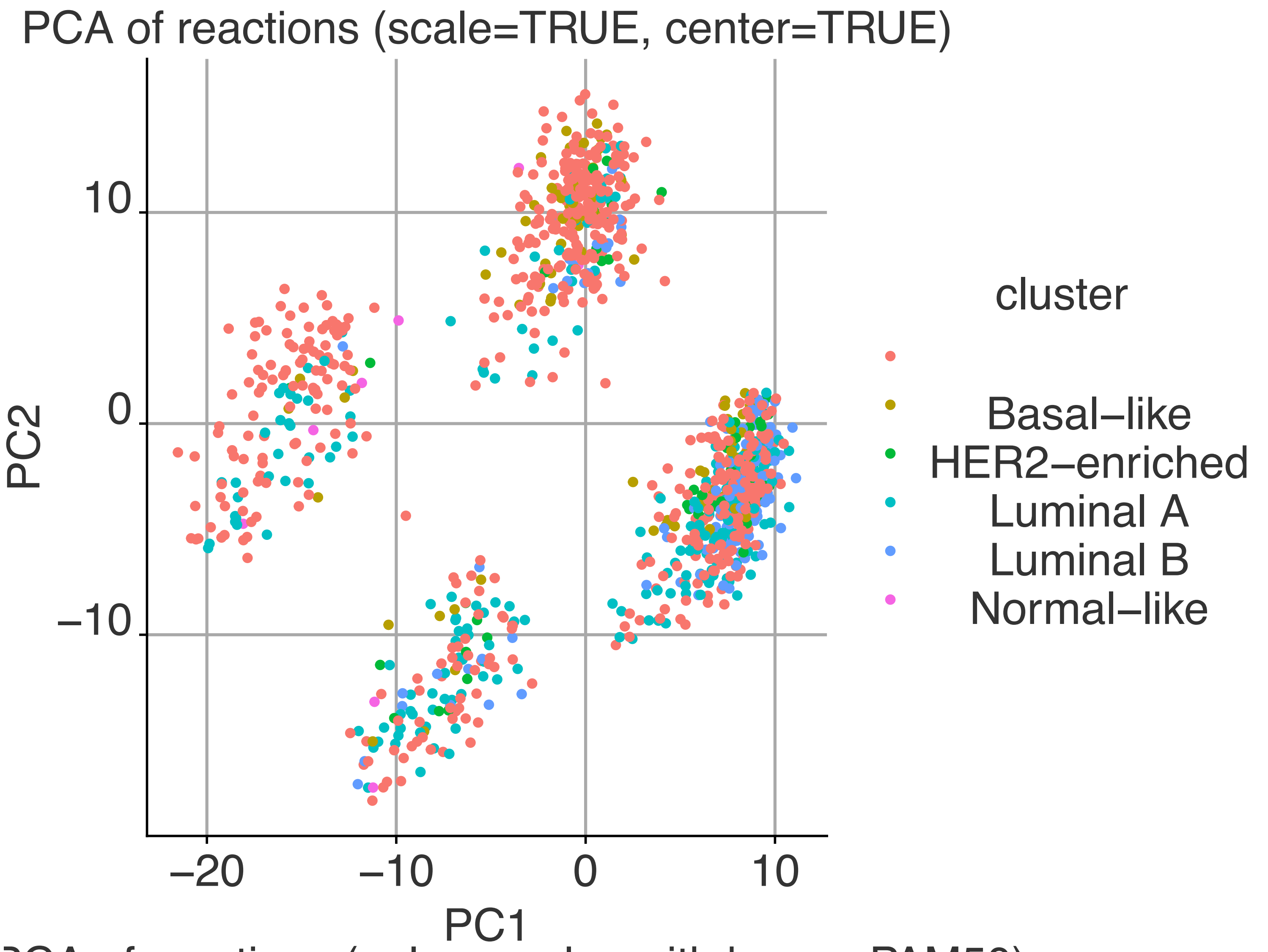

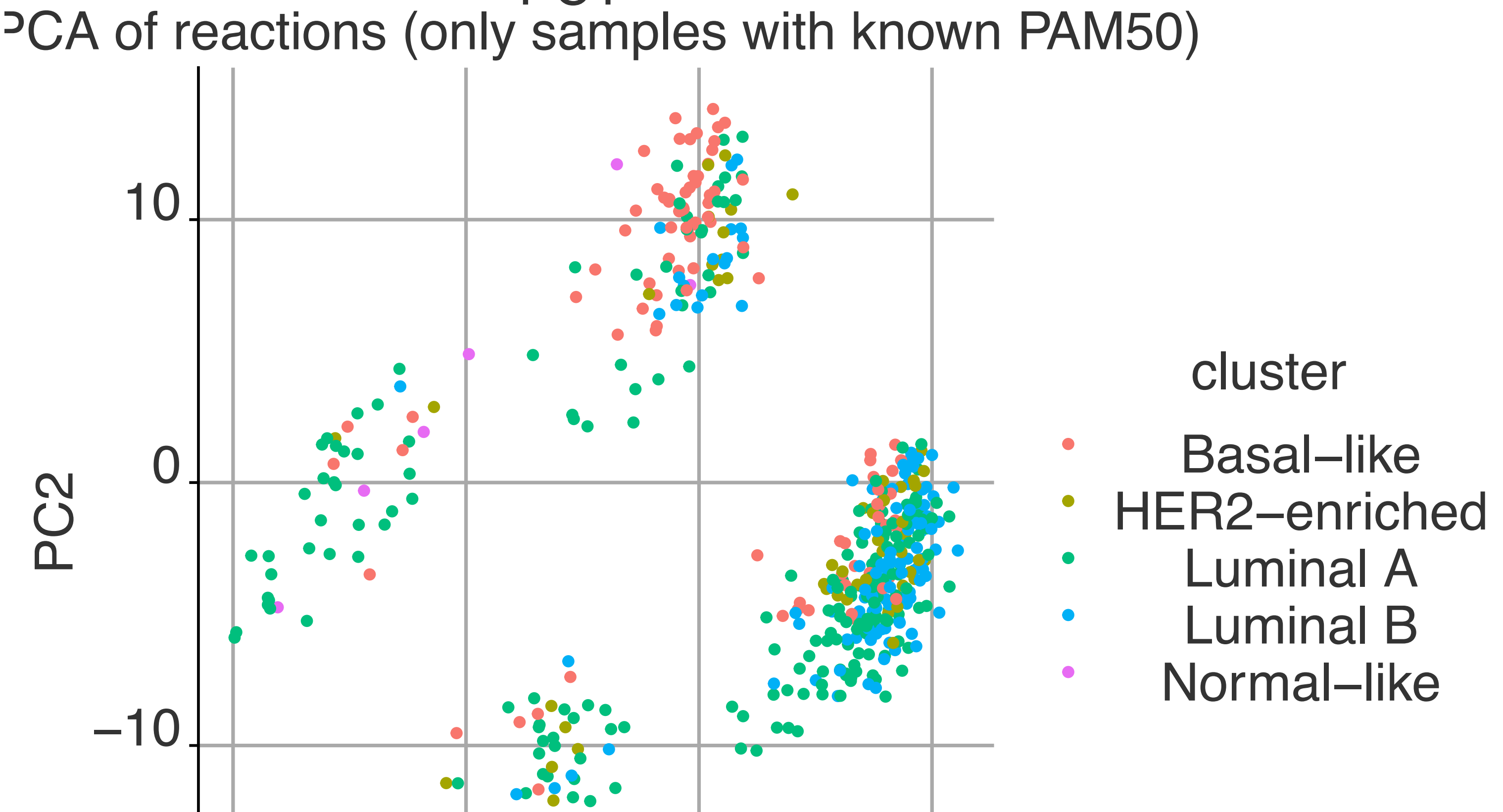

Table 1: Clusters by PAM50 (Chi-sq P=0.0000)

	1	2	3	4
	109	185	227	58
Basal-like	5	33	52	7
HER2-enriched	2	36	11	9
Luminal A	32	122	31	46
Luminal B	1	98	16	12
Normal-like	4	0	2	2

Table 2: Clusters by PAM50 as proportions for each subtype

	1	2	3	4
	0.19	0.32	0.39	0.10
Basal-like	0.05	0.34	0.54	0.07
HER2-enriched	0.03	0.62	0.19	0.16
Luminal A	0.14	0.53	0.13	0.20
Luminal B	0.01	0.77	0.13	0.09
Normal-like	0.50	0.00	0.25	0.25

4.2 ER Status

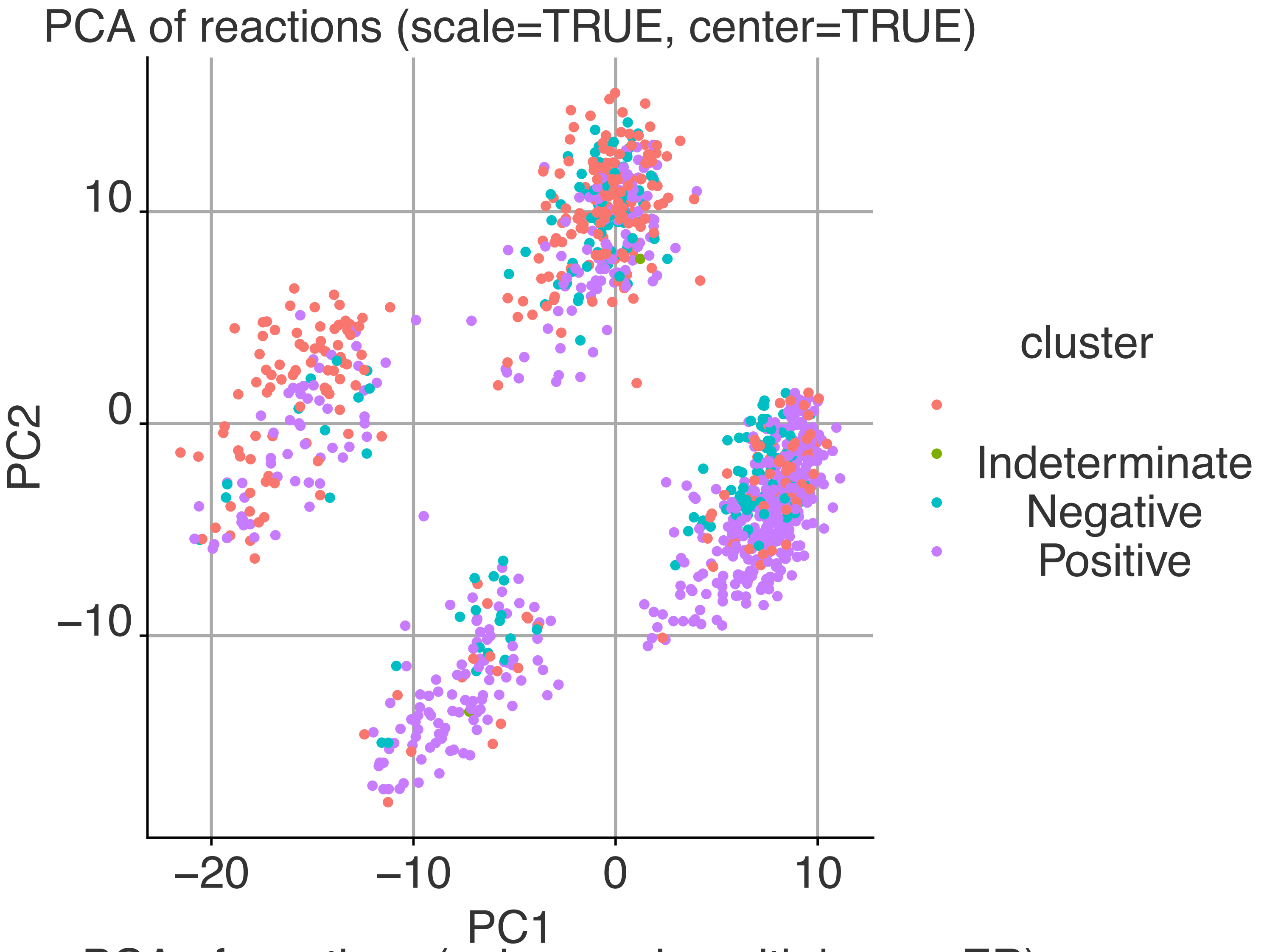
PCA of reactions (scale=TRUE, center=TRUE)
10
0
−10
−20
−10
0
10
PC1
PC2
cluster
Indeterminate
Negative
Positive

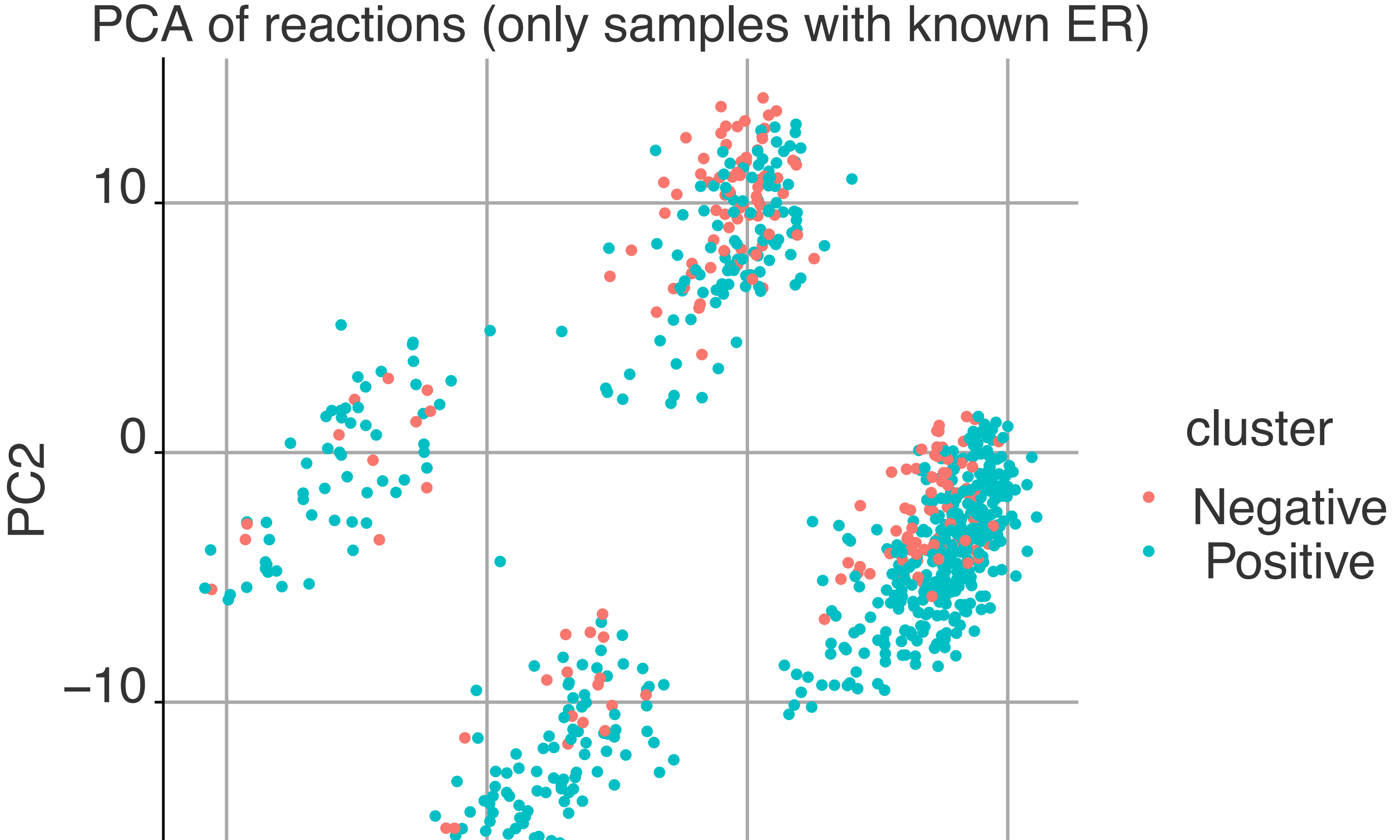
PCA of reactions (only samples with known ER)
10
0
−10
PC2
cluster
Negative
Positive

Table 3: Clusters by ER (Chi-sq P=0.0000)

	1	2	3	4
	84	62	160	16
Indeterminate	0	0	1	1
Negative	12	76	73	17
Positive	57	336	105	100

Table 4: Clusters by ER as proportions for each subtype

	1	2	3	4
	0.26	0.19	0.50	0.05
Indeterminate	0.00	0.00	0.50	0.50
Negative	0.07	0.43	0.41	0.10
Positive	0.10	0.56	0.18	0.17

4.3 Triple Negative Status

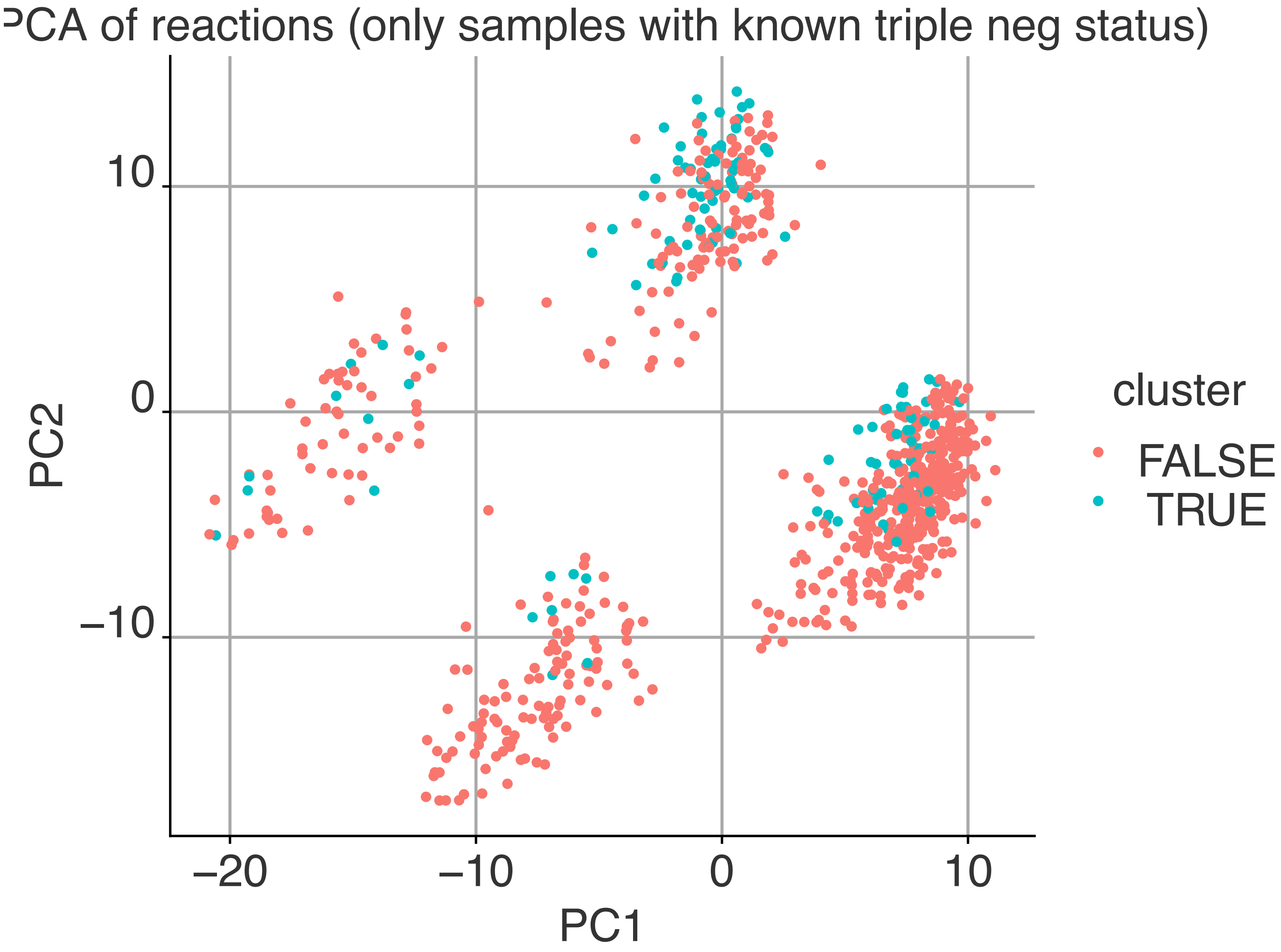

Table 5: Clusters by triple neg (Chi-sq P=0.0000)

	1	2	3	4
FALSE	58	362	115	110
TRUE	10	47	58	7

Table 6: Clusters by triple neg as proportions

	1	2	3	4
FALSE	0.09	0.56	0.18	0.17
TRUE	0.08	0.39	0.48	0.06

4.4 AJCC Stage

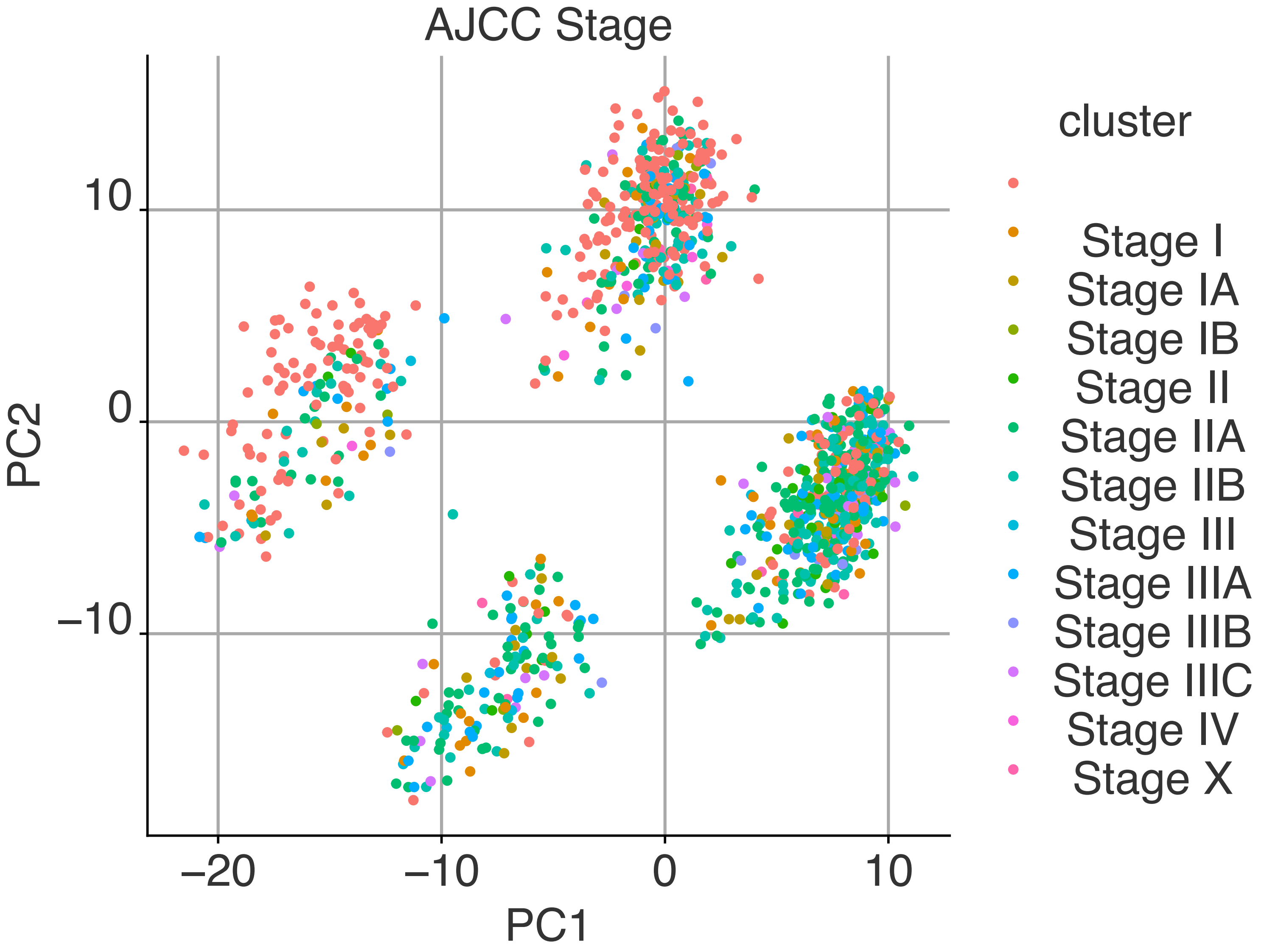

4.5 Percent lymphocyte infiltration

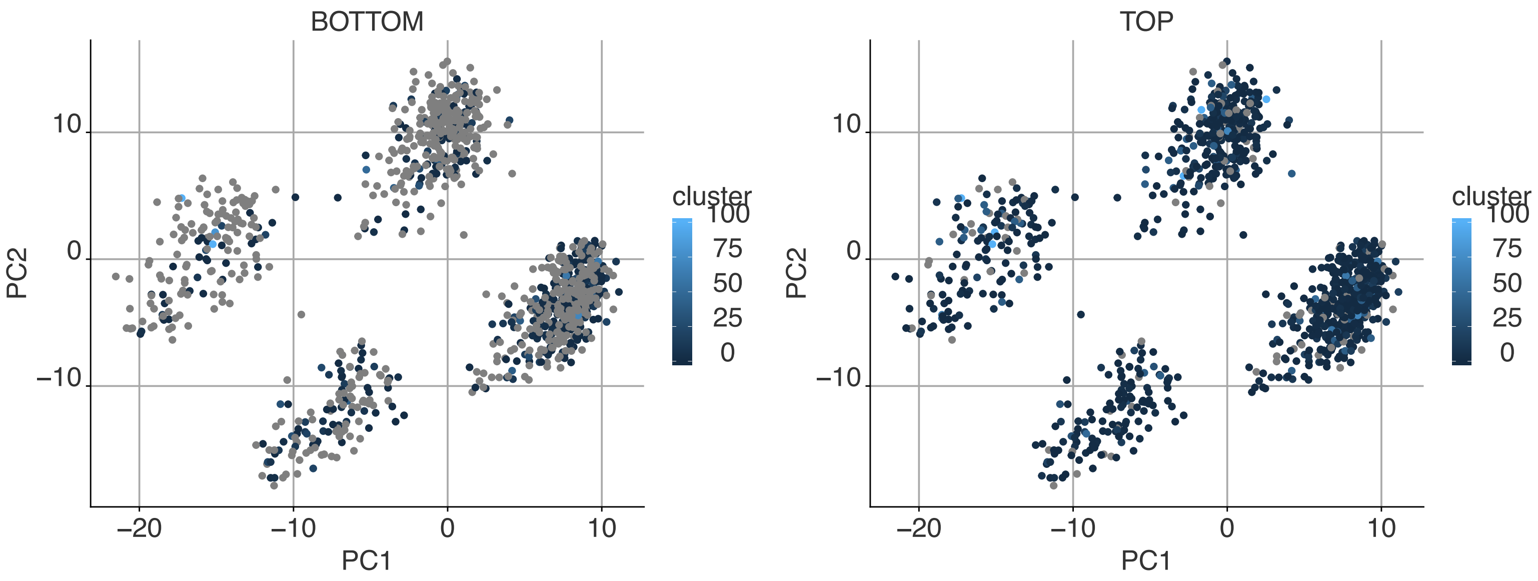

4.6 Percent monocyte infiltration

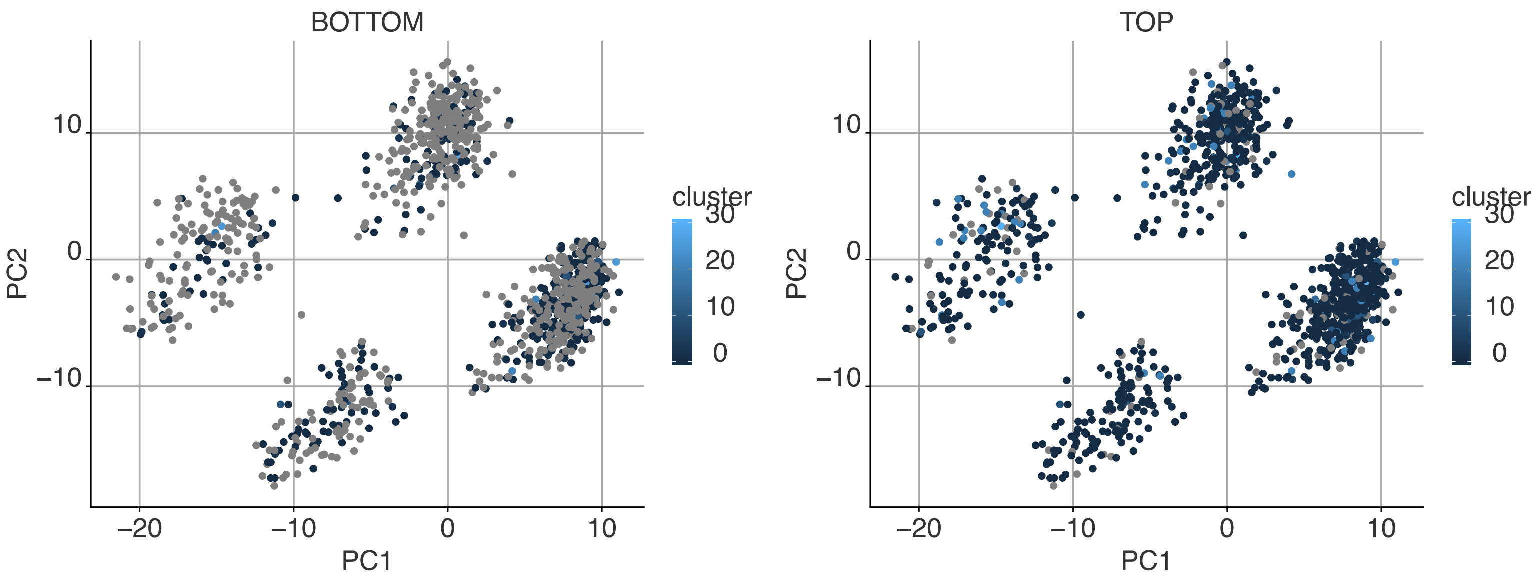

4.7 Percent necrosis

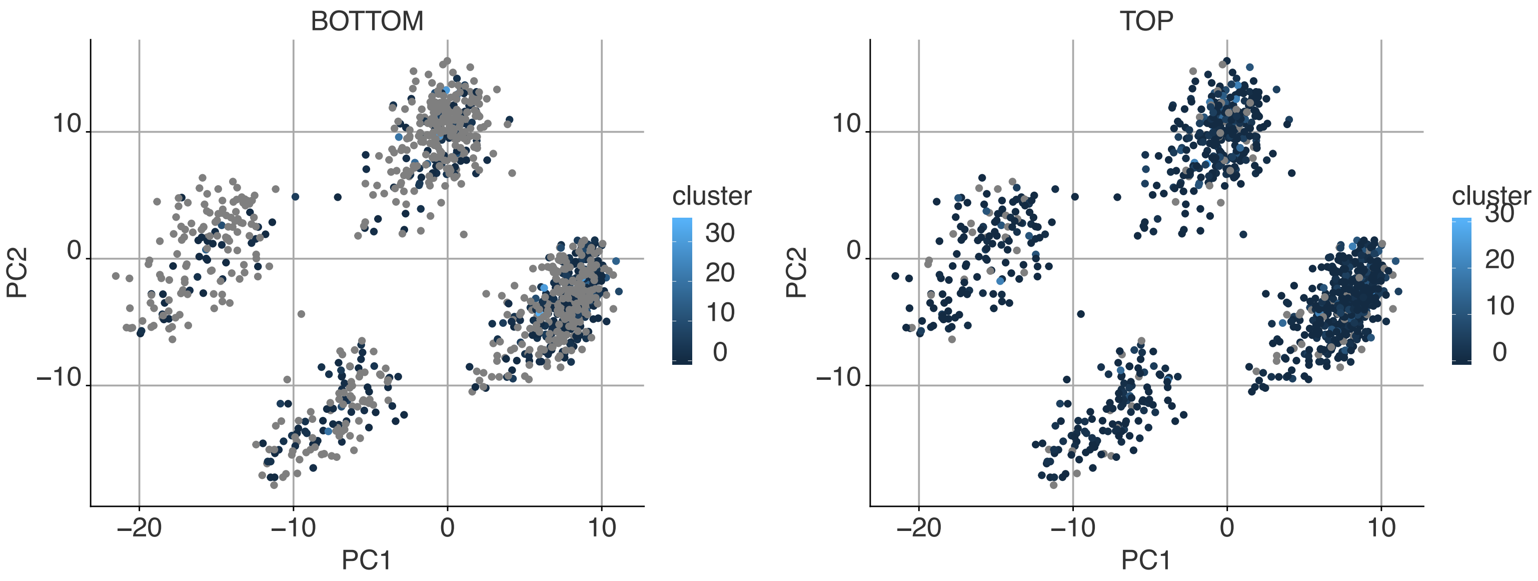

4.8 Percent neutrophil infiltration

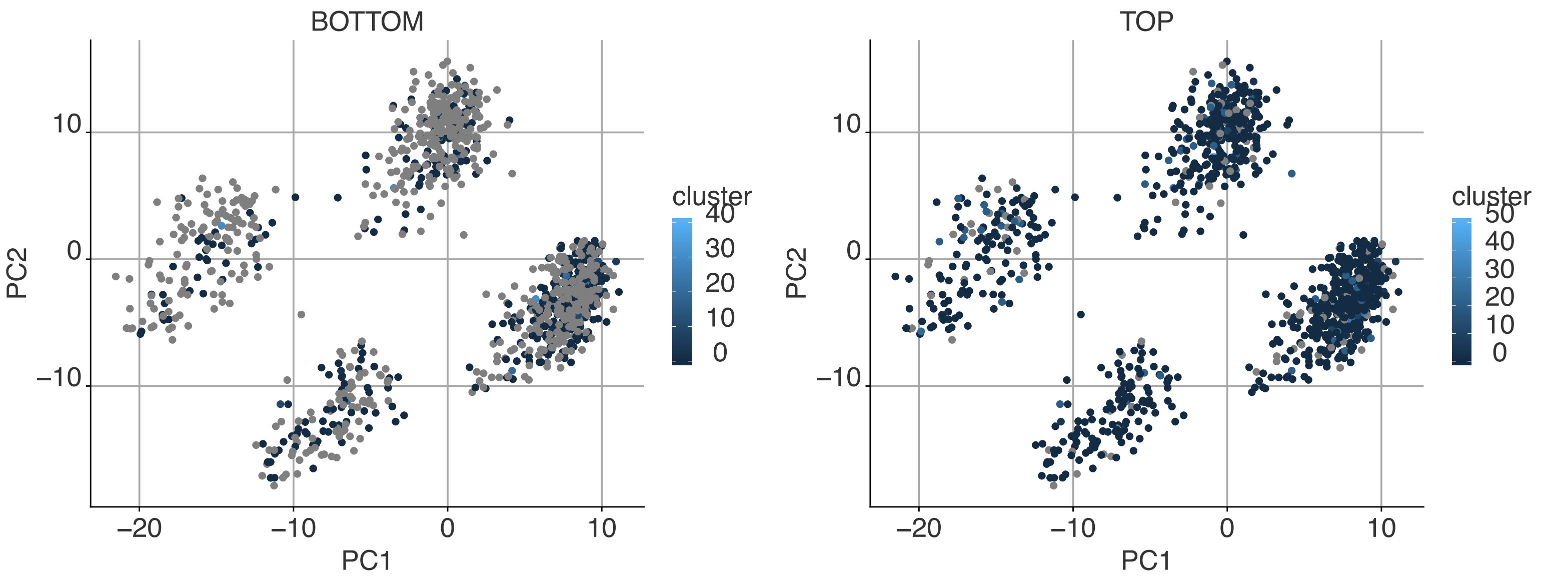

4.9 Percent normal cells

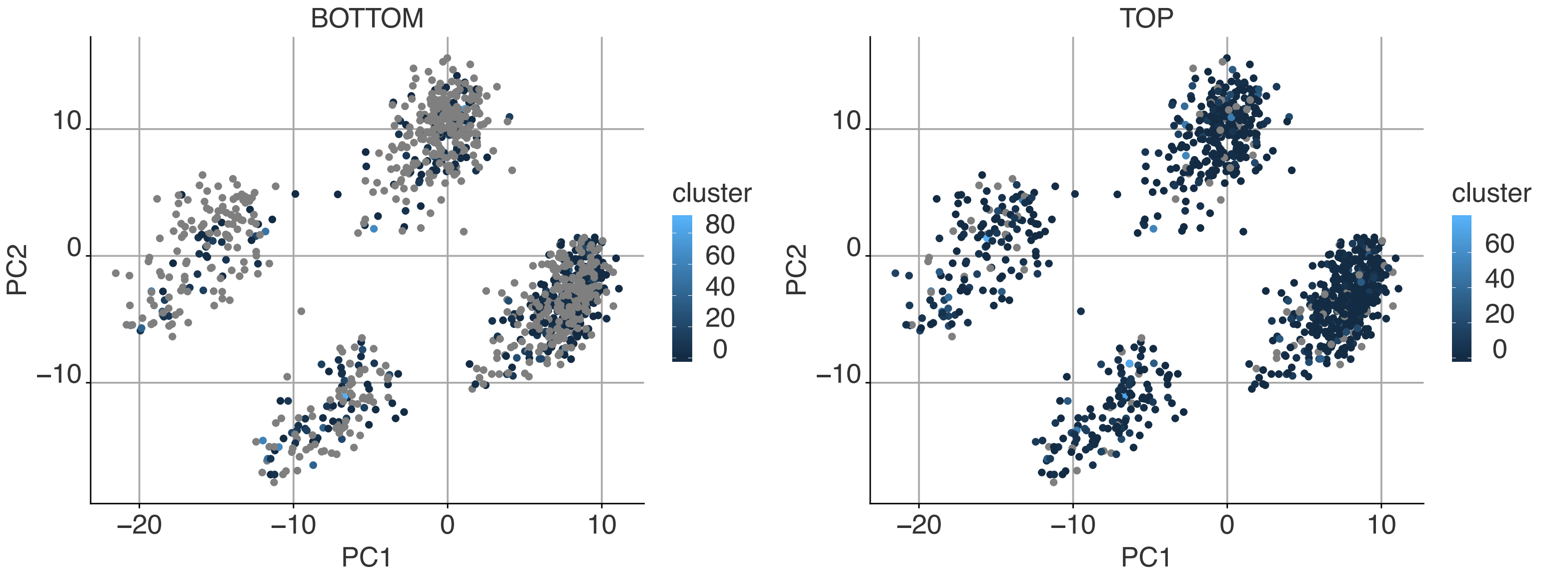

4.10 Percent stromal cells

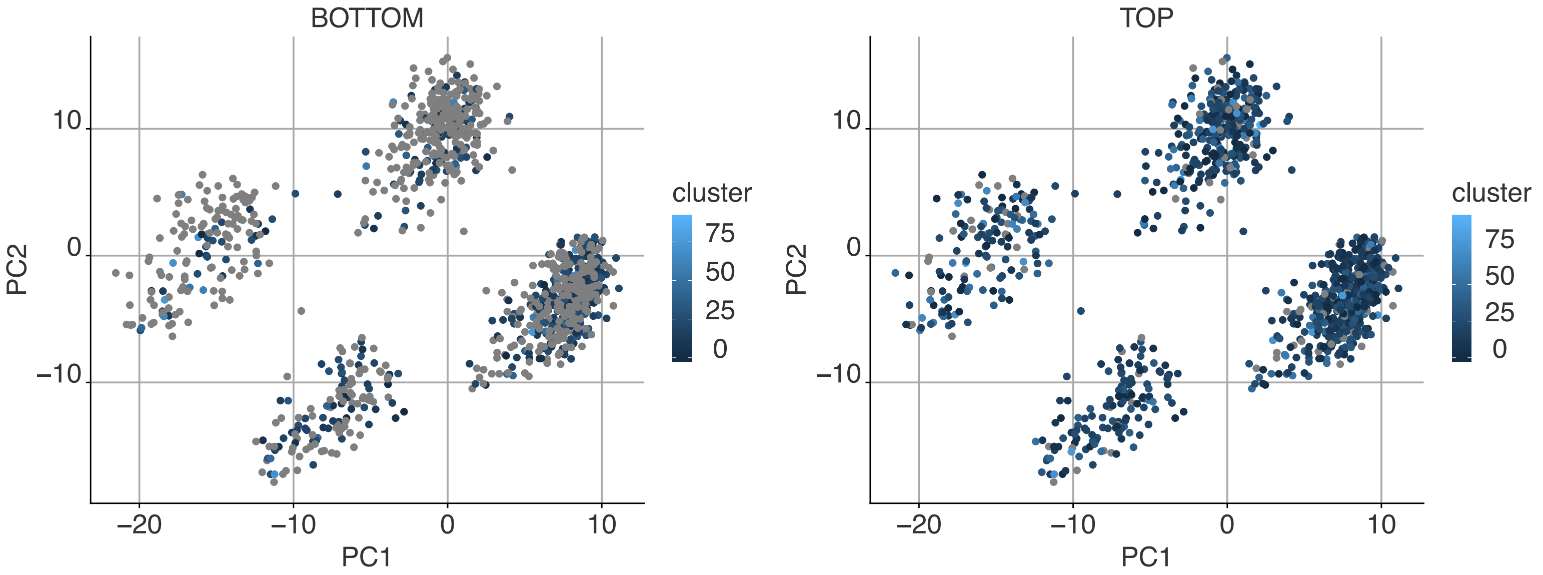

4.11 Percent tumor cells

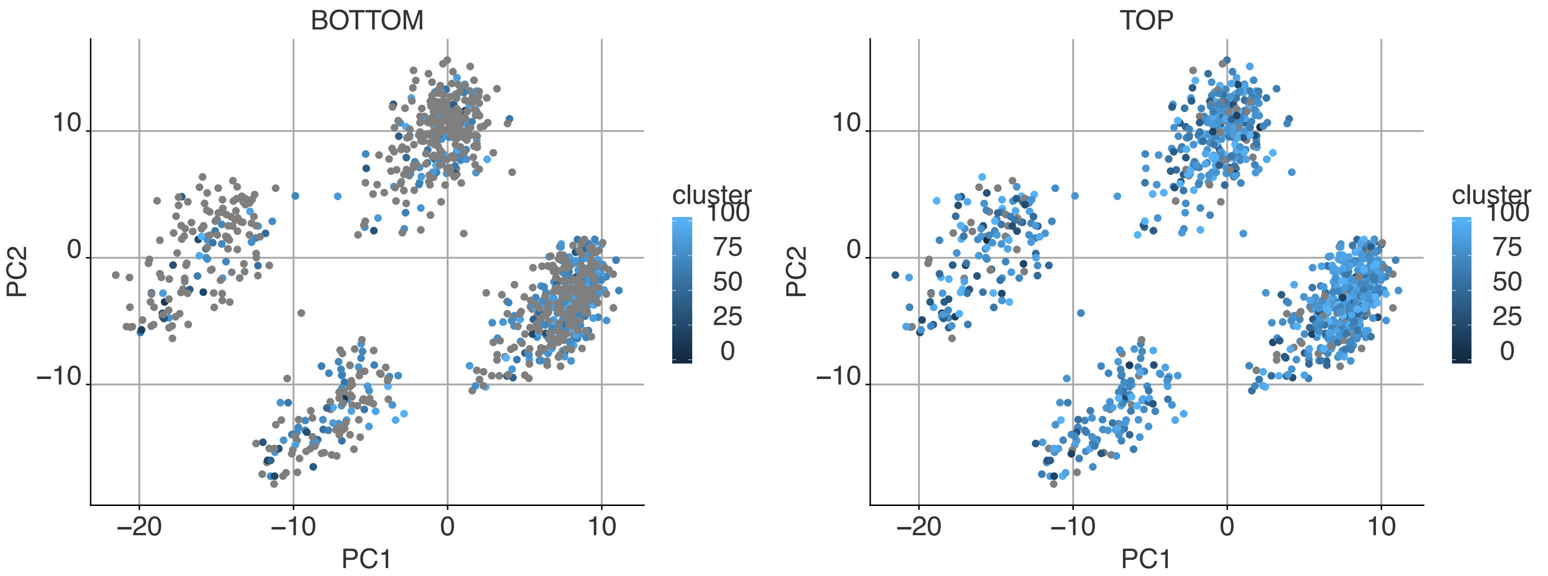

4.12 Tissue source site

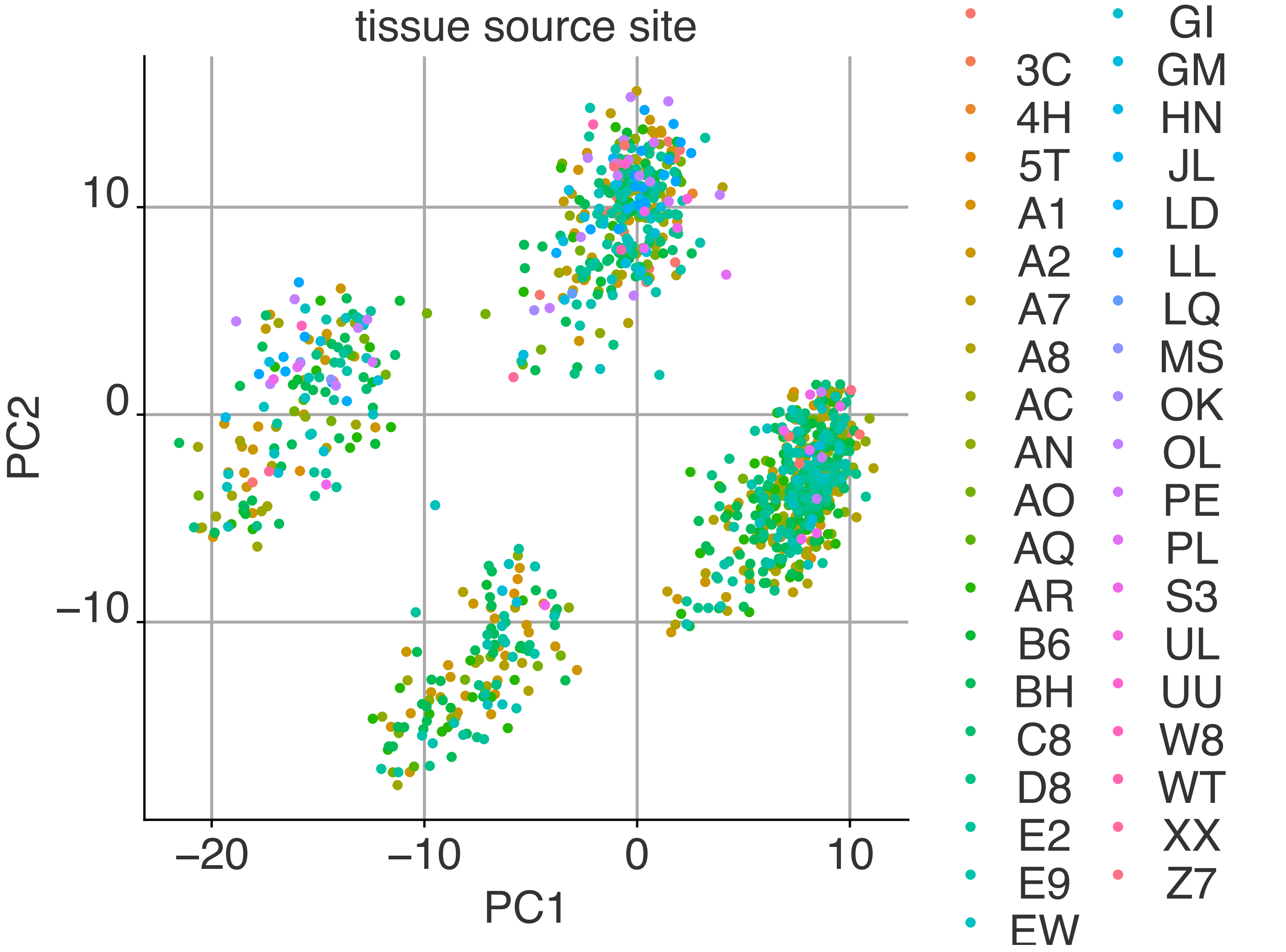

4.13 Year of initial pathologic diagnosis

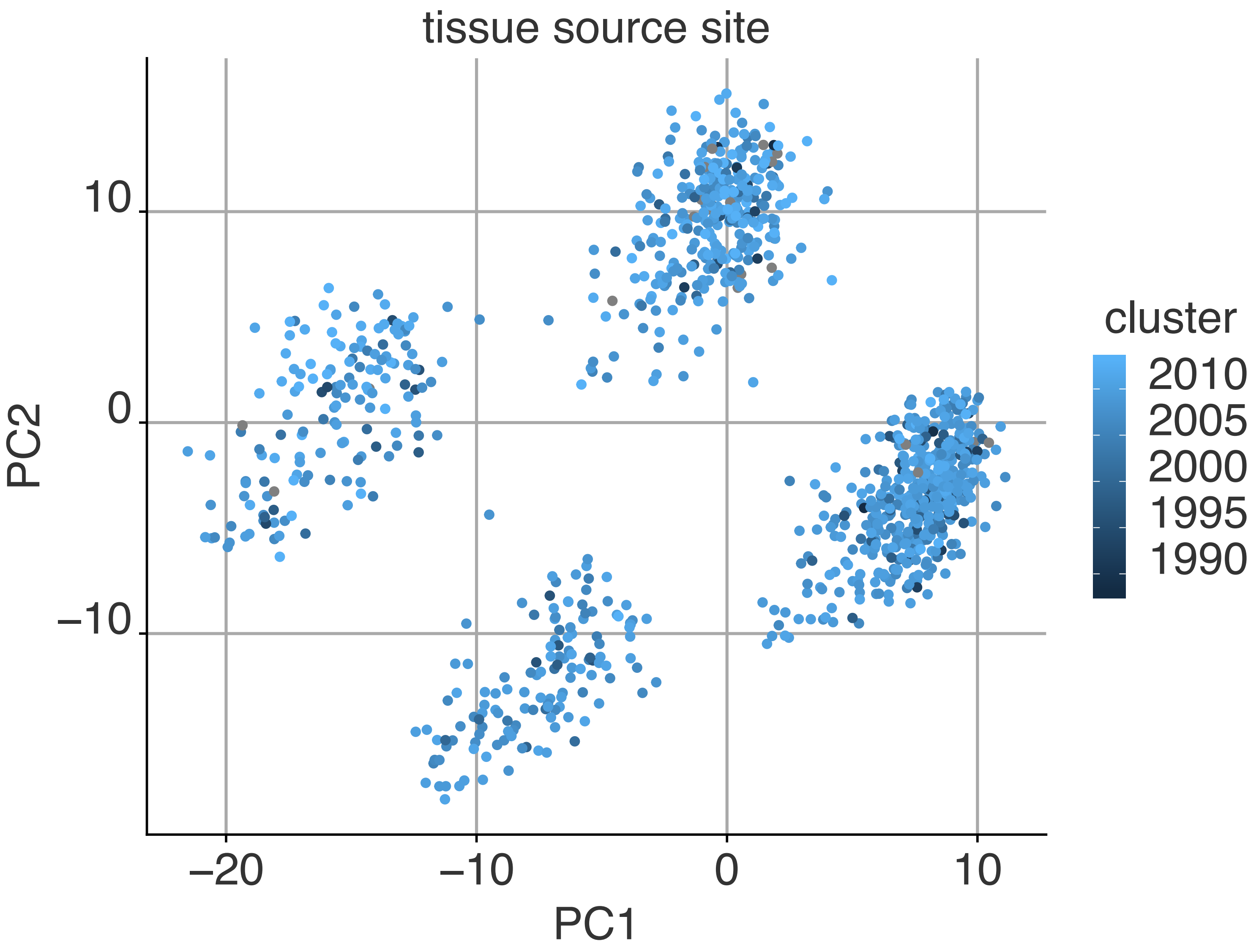

4.14 Pathologic state

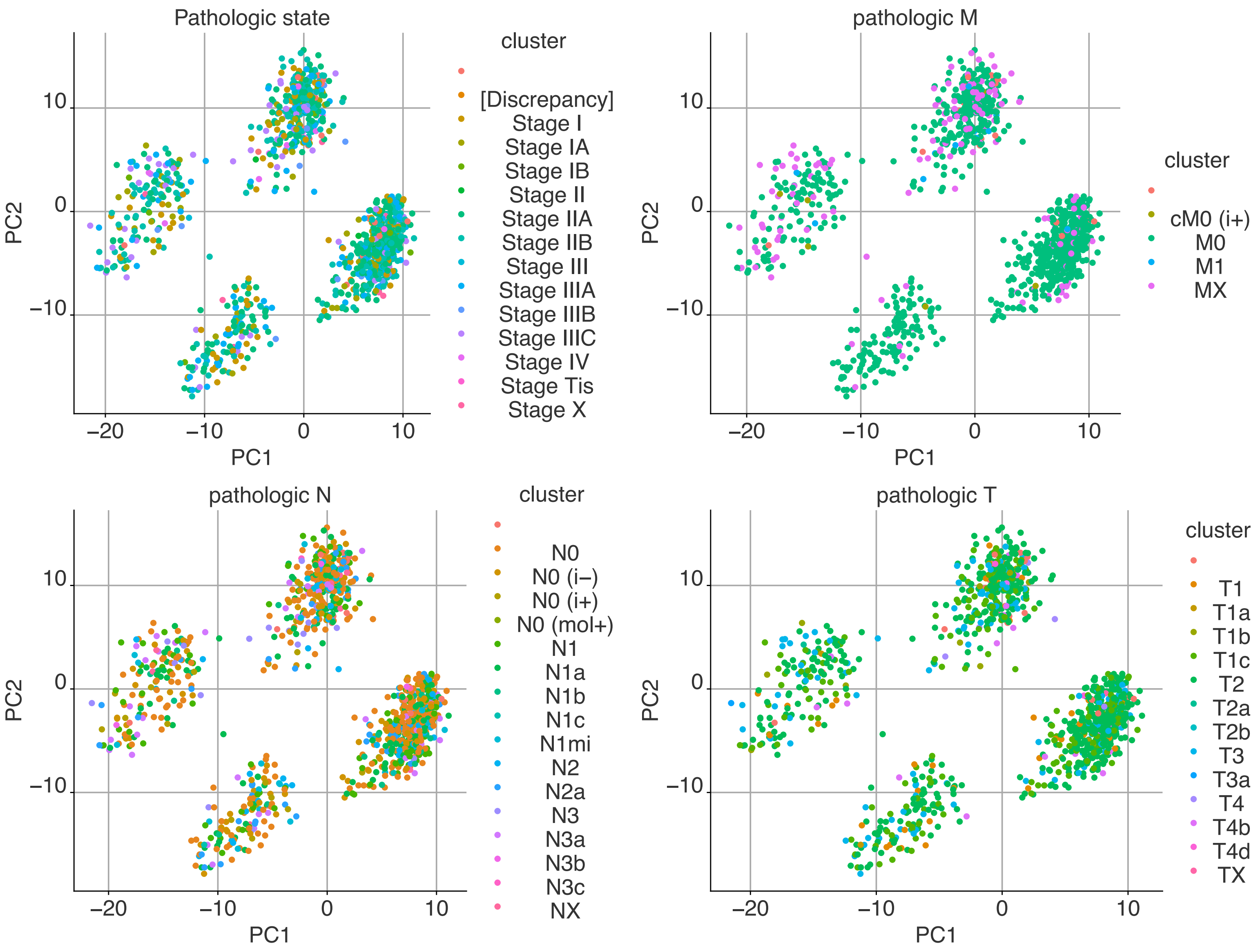

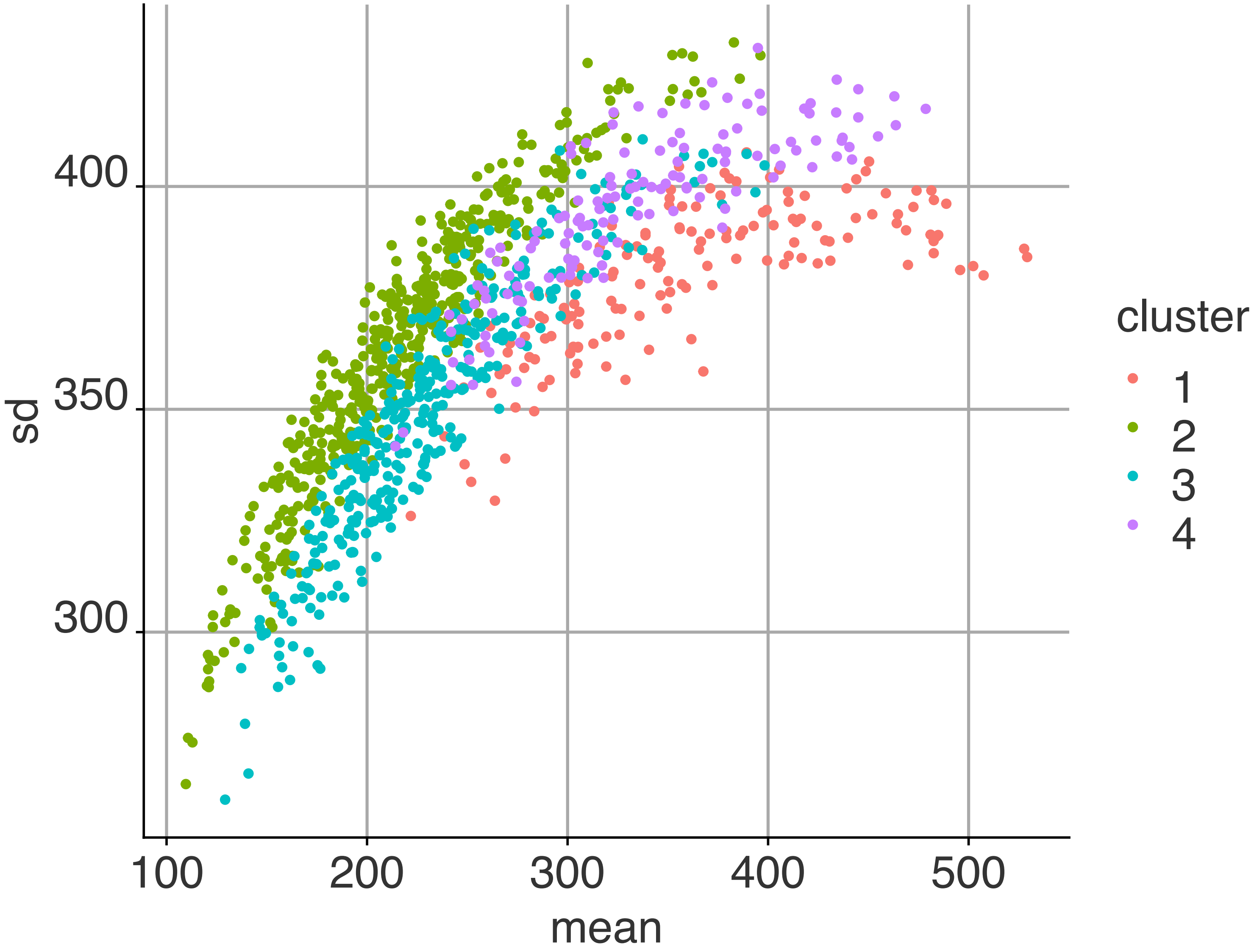

For 6 randomly selected samples we plot the densities of the reactions:

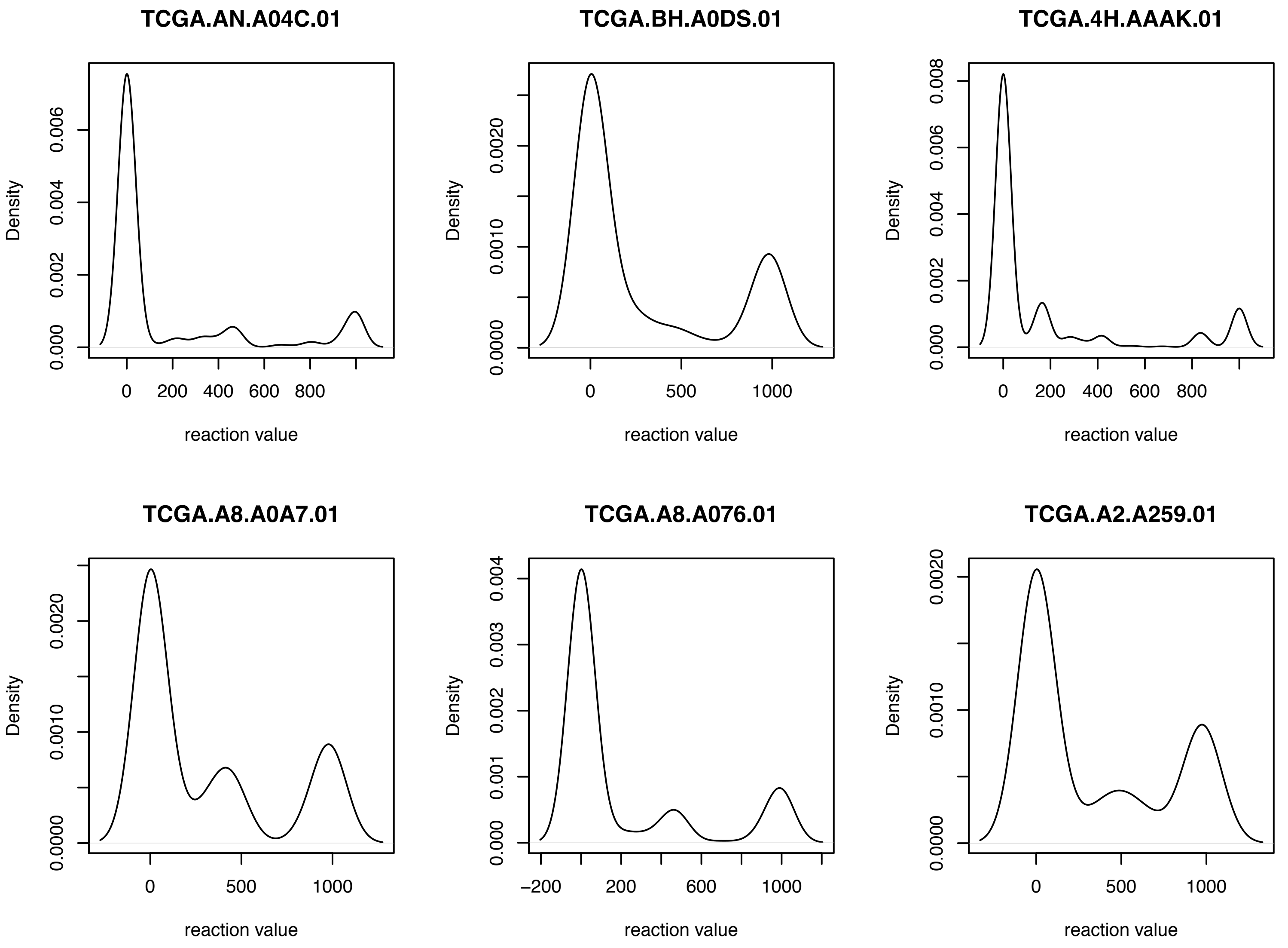

Here we draw the densities of 6 randomly selected reactions:

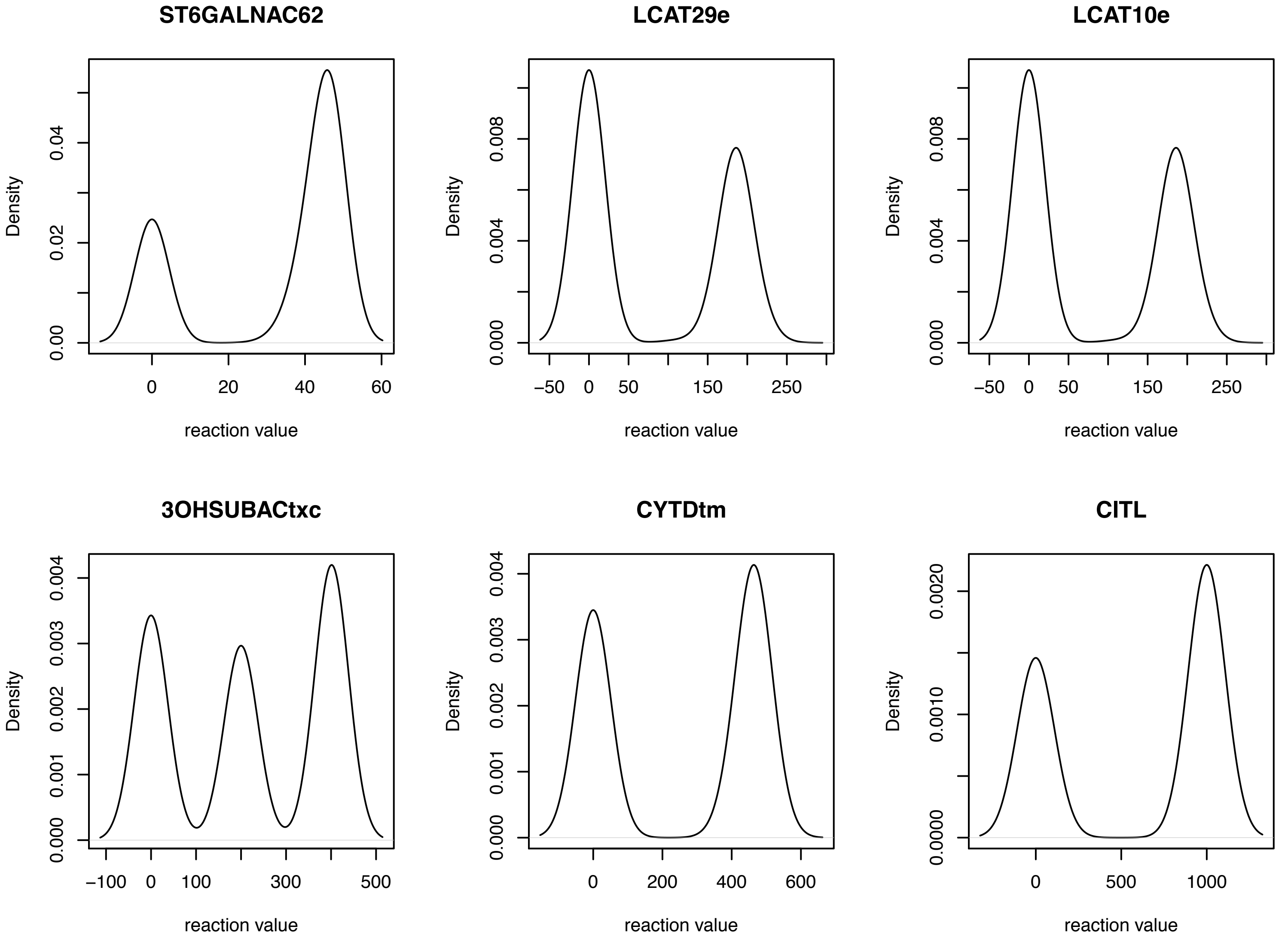

Given that reactions have a zero and a non-zero state, we convert the reaction data to binary (less than $10 = 0$, 1 otherwise) and compute a χ^2 test between the four clusters.

	statistic	pval	pbon
17AHPRGNLONEte	1100	0	0
ACMPtu	1100	0	0
AHANDROSTANte	1100	0	0
ATVLACtu	1100	0	0
C03681te	1100	0	0
C05299te	1100	0	0
C05301te	1100	0	0
C05302te	1100	0	0
CE2211te	1100	0	0
CE5072te	1100	0	0
CSAtd	1100	0	0
CSAtu	1100	0	0
EX__17ahprgnlone[e]	1100	0	0
EX__C03681[e]	1100	0	0
EX__C05299[e]	1100	0	0
EX__C05301[e]	1100	0	0
EX__C05302[e]	1100	0	0
EX__CE2211[e]	1100	0	0
EX__CE5072[e]	1100	0	0
EX__prgnlone[e]	1100	0	0
EX__prgnlones[e]	1100	0	0
LSTNtu	1100	0	0
LVSTPGPtu	1100	0	0
LVSTtu	1100	0	0

	statistic	pval	pbon
MDZtd	1100	0	0
MDZtu	1100	0	0
PRGNLONESte	1100	0	0
PRGNLONEte	1100	0	0
RE1587C	1100	0	0
RE1630C	1100	0	0

We find that many reactions have identical states among samples when binarized (i.e. they are zero in the same samples and non-zero in the same samples). For example, when ranked with the above χ^2 test, the first 38 reactions have the same zero/non-zero states and the next 44 reactions have the same states.

This is the very first reaction (the first 38 reactions have the same test statistics) after ranking with the χ^2 test:

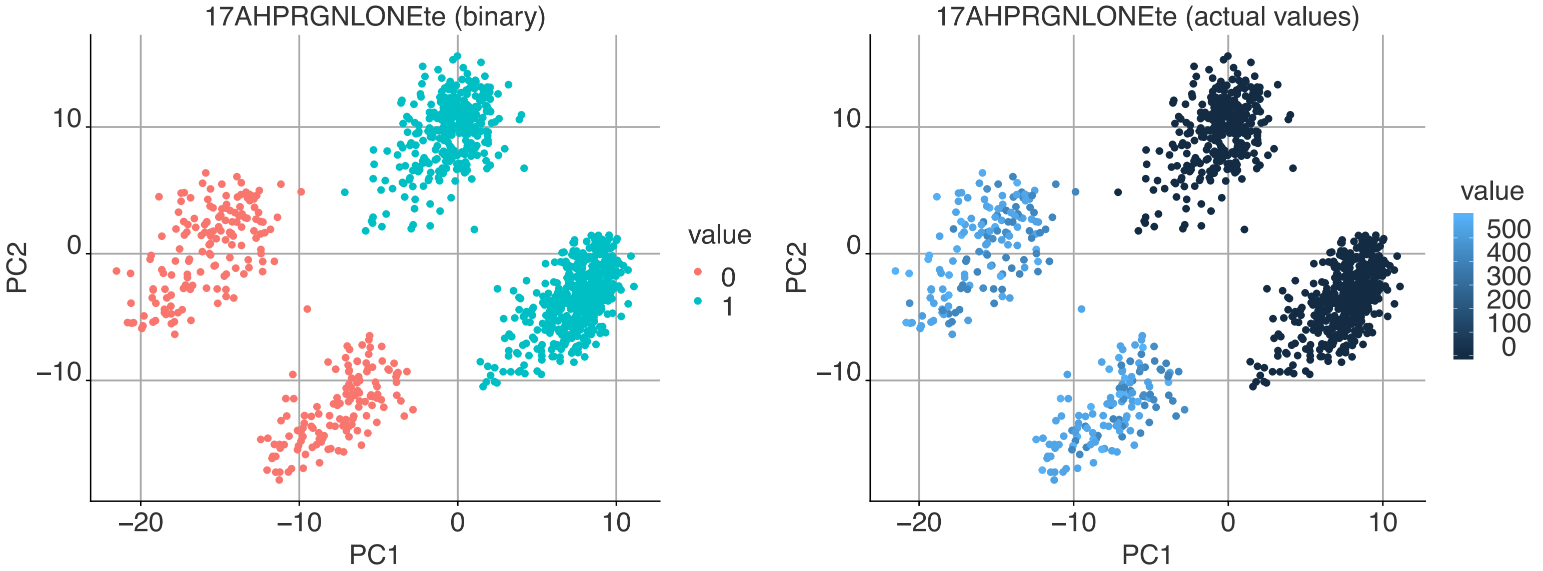

This is the 39th ranking reaction:

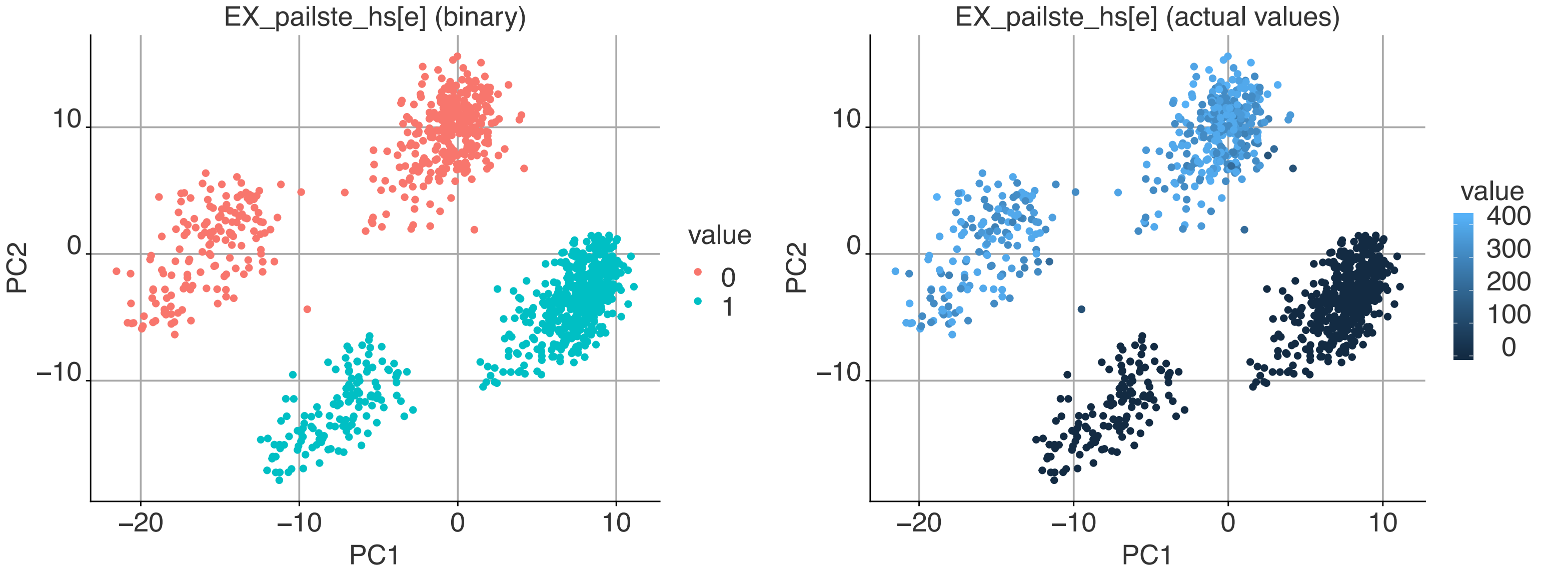

100th ranking reaction:

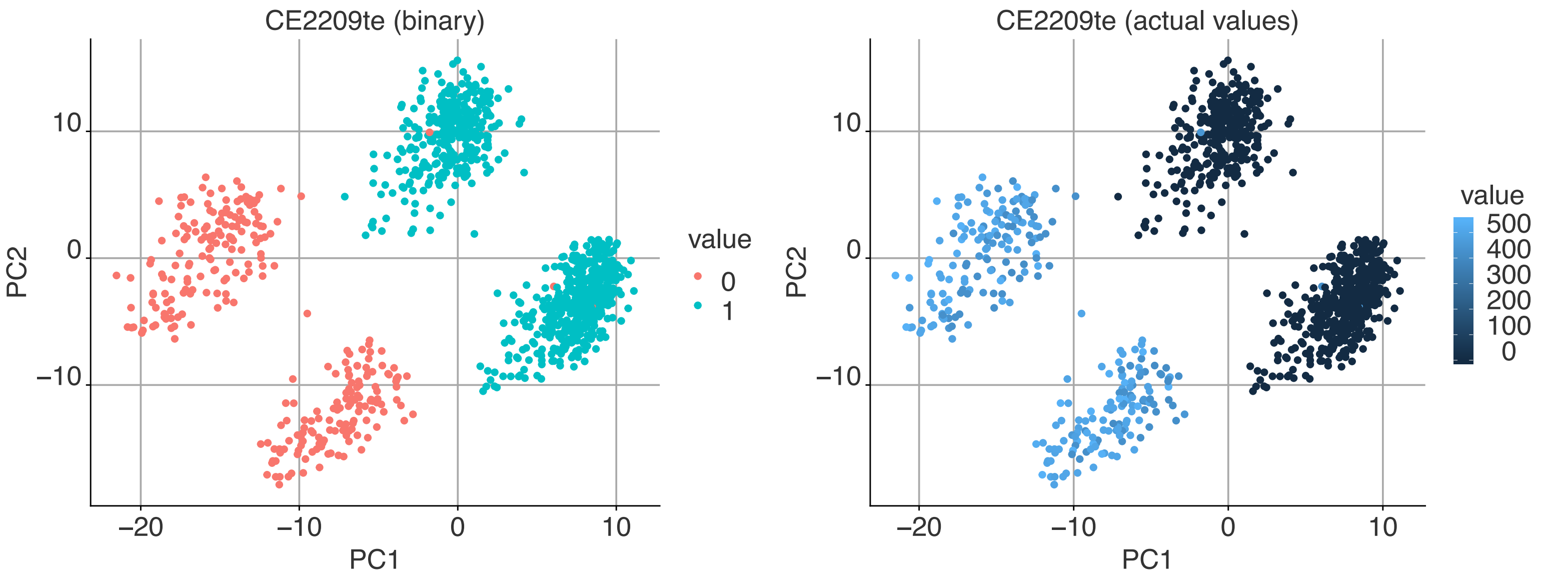

110th ranking reaction:

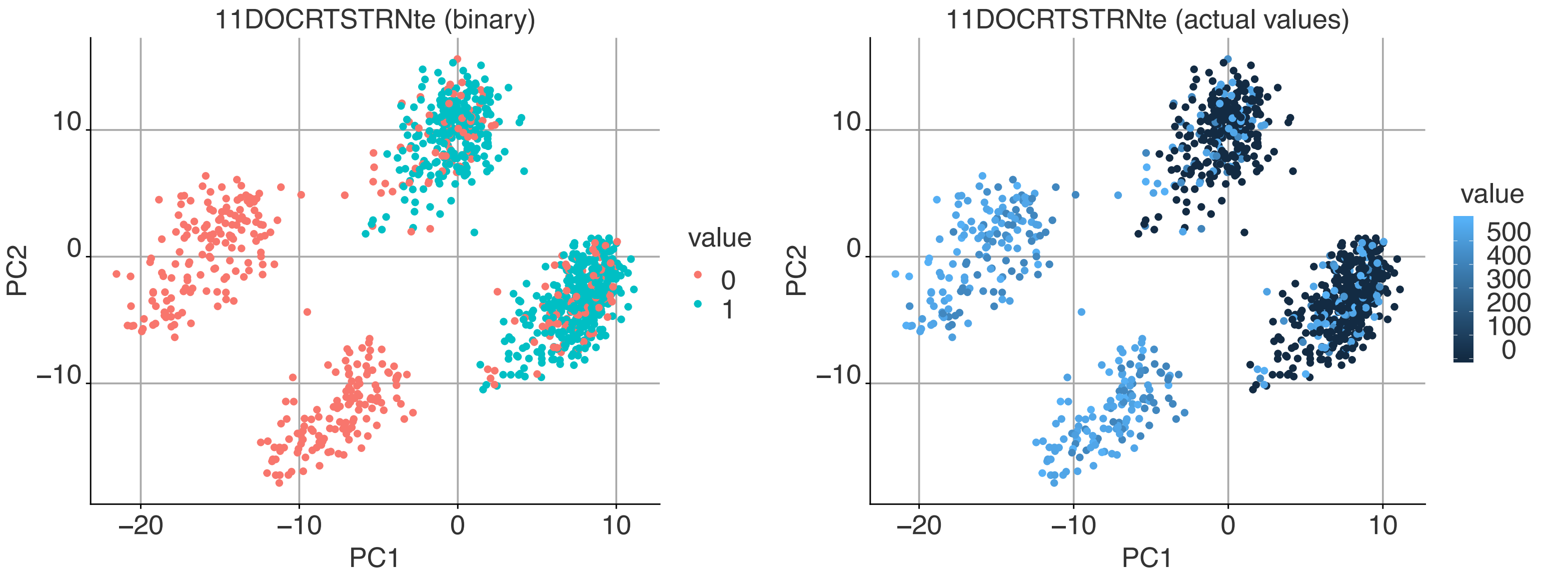

150th ranking reaction:

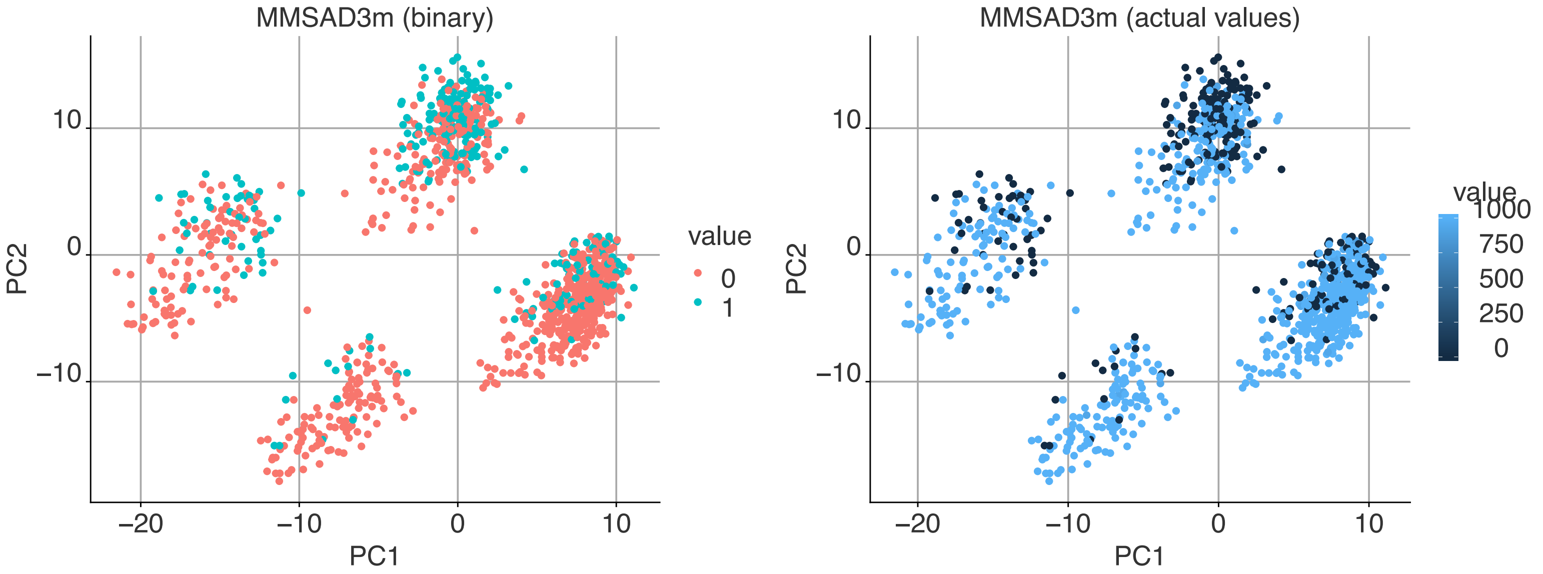

6 Analysis of divergence data

We next perform a χ^s test with the divergence values for the 4 clusters.

	statistic	pval	pbon
ABCB1	1100.0000	0	0
LCAT	1097.7115	0	0
DARC	456.6299	0	0
FREM1	425.6843	0	0
S1PR1	398.4353	0	0
EMCN	356.8341	0	0
MEOX1	355.6835	0	0
SELP	369.6766	0	0
TSPAN7	352.5677	0	0
DNASE1L3	330.7277	0	0
MMRN2	327.3382	0	0
CCL14	322.8991	0	0
GIPC2	322.6501	0	0
RASGRP2	315.3382	0	0
NRIP2	312.2394	0	0
LCN10	305.7732	0	0
ADCY4	305.7314	0	0
SCN3A	301.8932	0	0
TNXB	287.4456	0	0
GIMAP8	300.2883	0	0
CADM3	299.5878	0	0
GRRP1	298.7082	0	0
PEAR1	284.3775	0	0
TSHZ2	297.6442	0	0
AVPR2	281.5979	0	0
TGFBR2	293.6934	0	0
CXorf36	293.3908	0	0
INMT	278.8178	0	0
LDB2	274.6064	0	0
ABCA10	274.0227	0	0

We plot the clusters and color the samples by divergence state and expression (TPM) value for some of the top ranking genes:

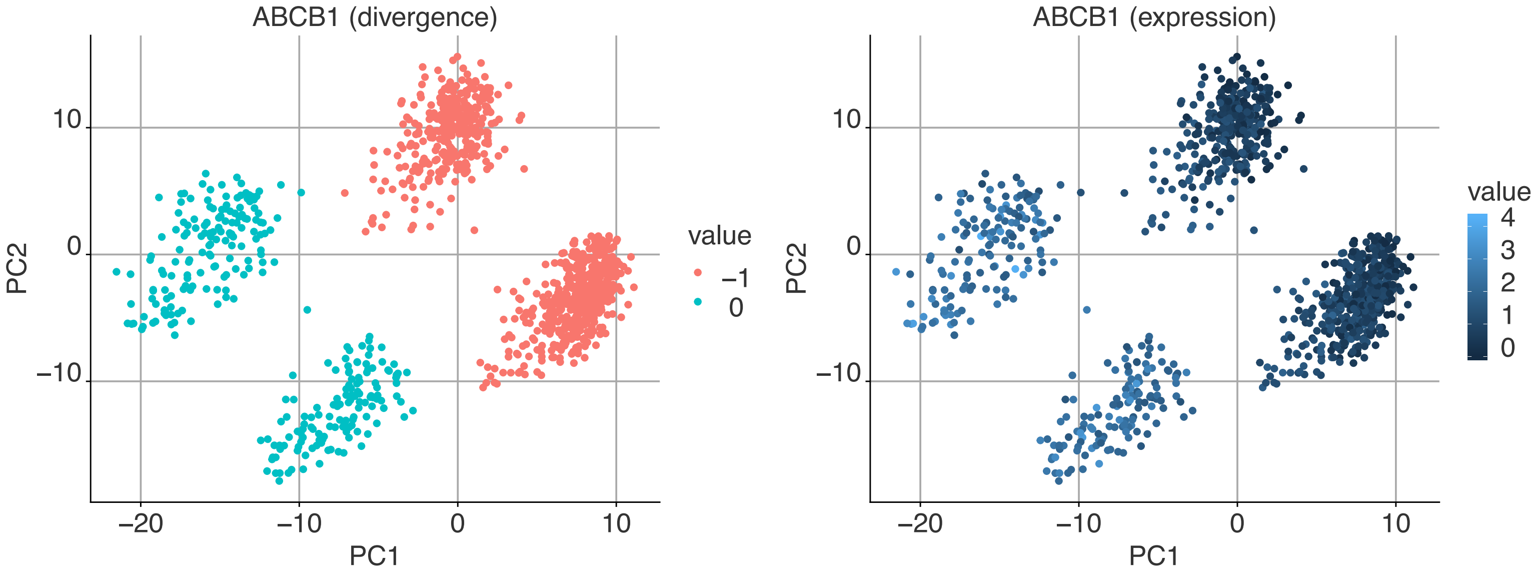

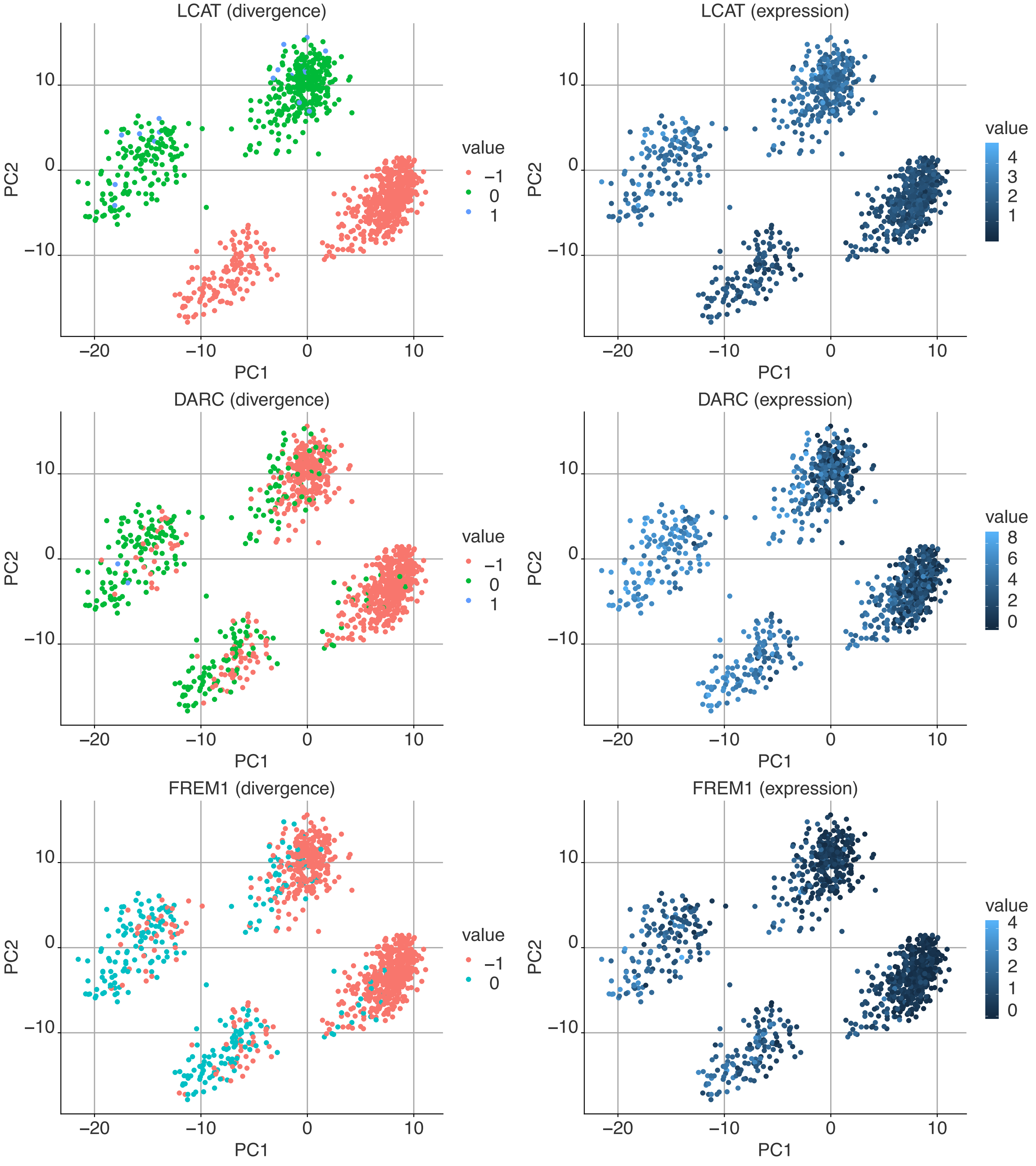

LCAT (divergence)
LCAT (expression)
DARC (divergence)
DARC (expression)
FREM1 (divergence)
FREM1 (expression)
PC1
PC2
−20
−10
0
10
value
−1
0
1
4
3
2
1
8
6
4
2
0

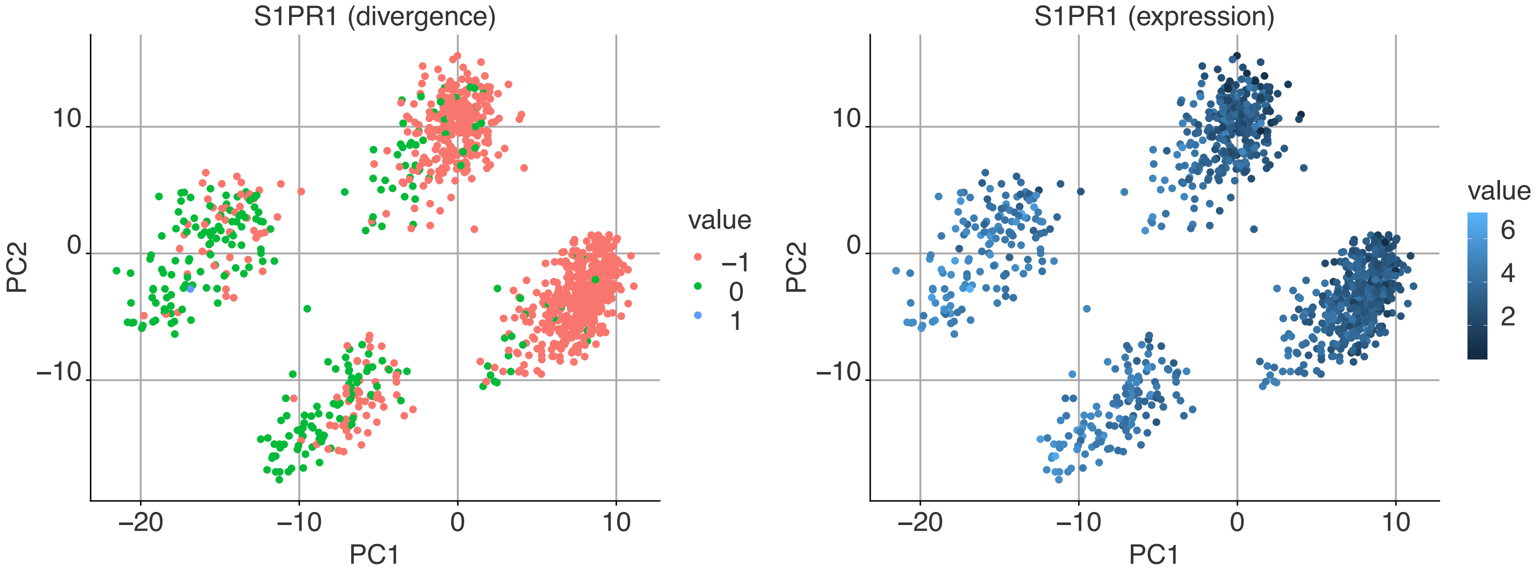

7 Molecular Checks

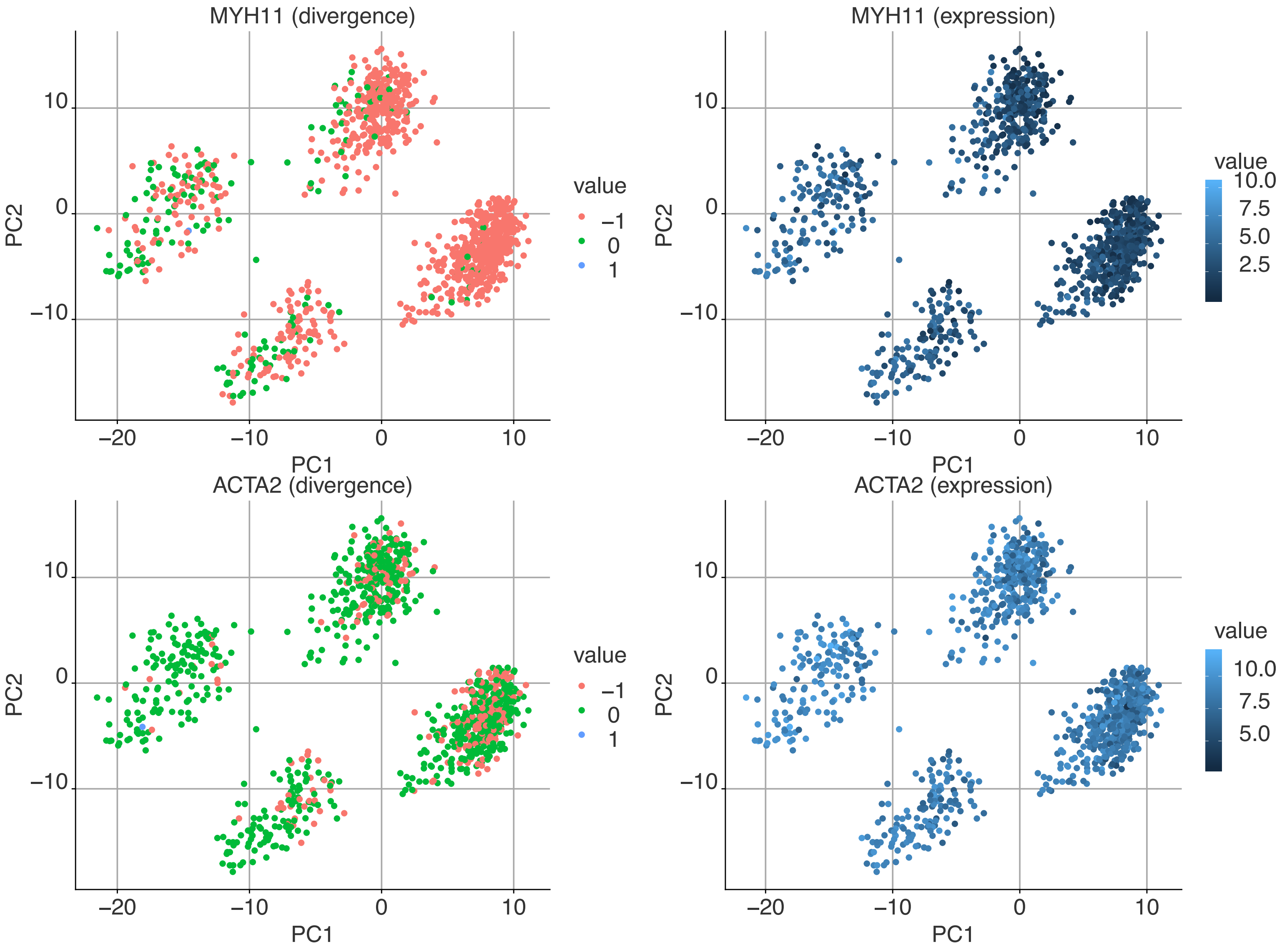

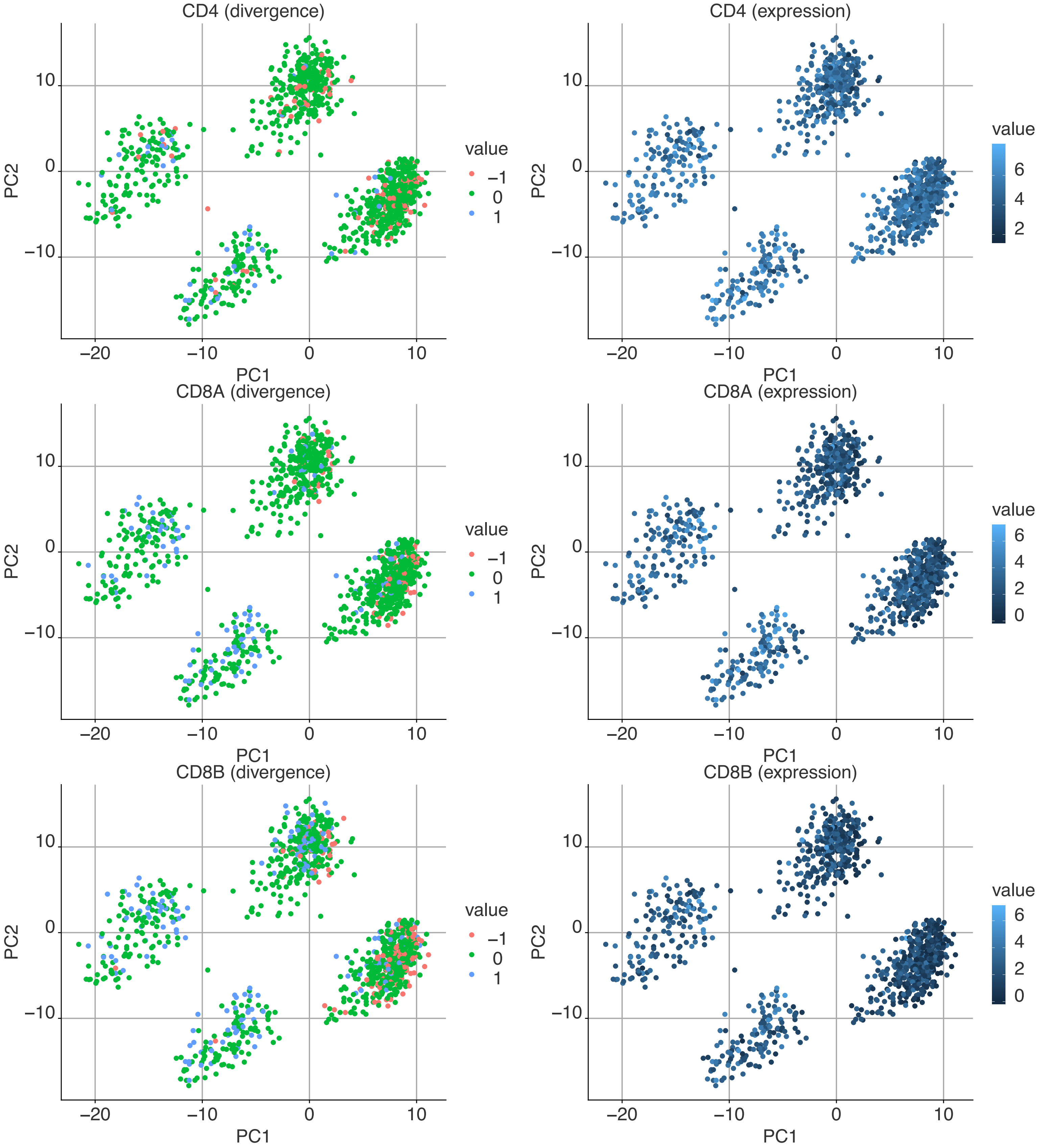

CD4 (divergence)
CD4 (expression)
CD8A (divergence)
CD8A (expression)
CD8B (divergence)
CD8B (expression)
PC1
PC2
−20
−10
0
10
value
−1
0
1
2
4
6

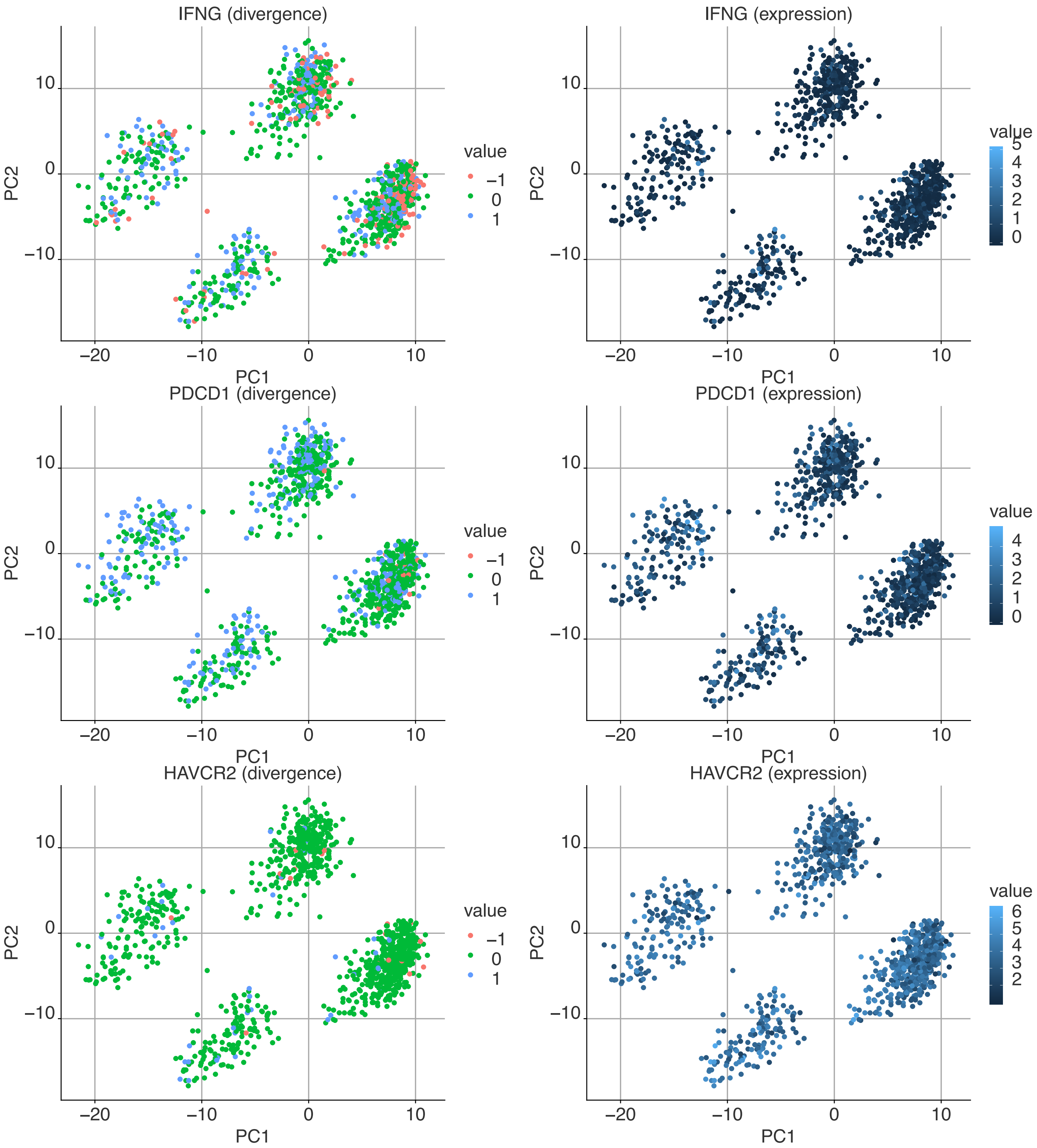
IFNG (divergence)
IFNG (expression)
PDCD1 (divergence)
PDCD1 (expression)
HAVCR2 (divergence)
HAVCR2 (expression)
PC1
PC2
value
−1
0
1
10
0
−10
−20
5
4
3
2
6

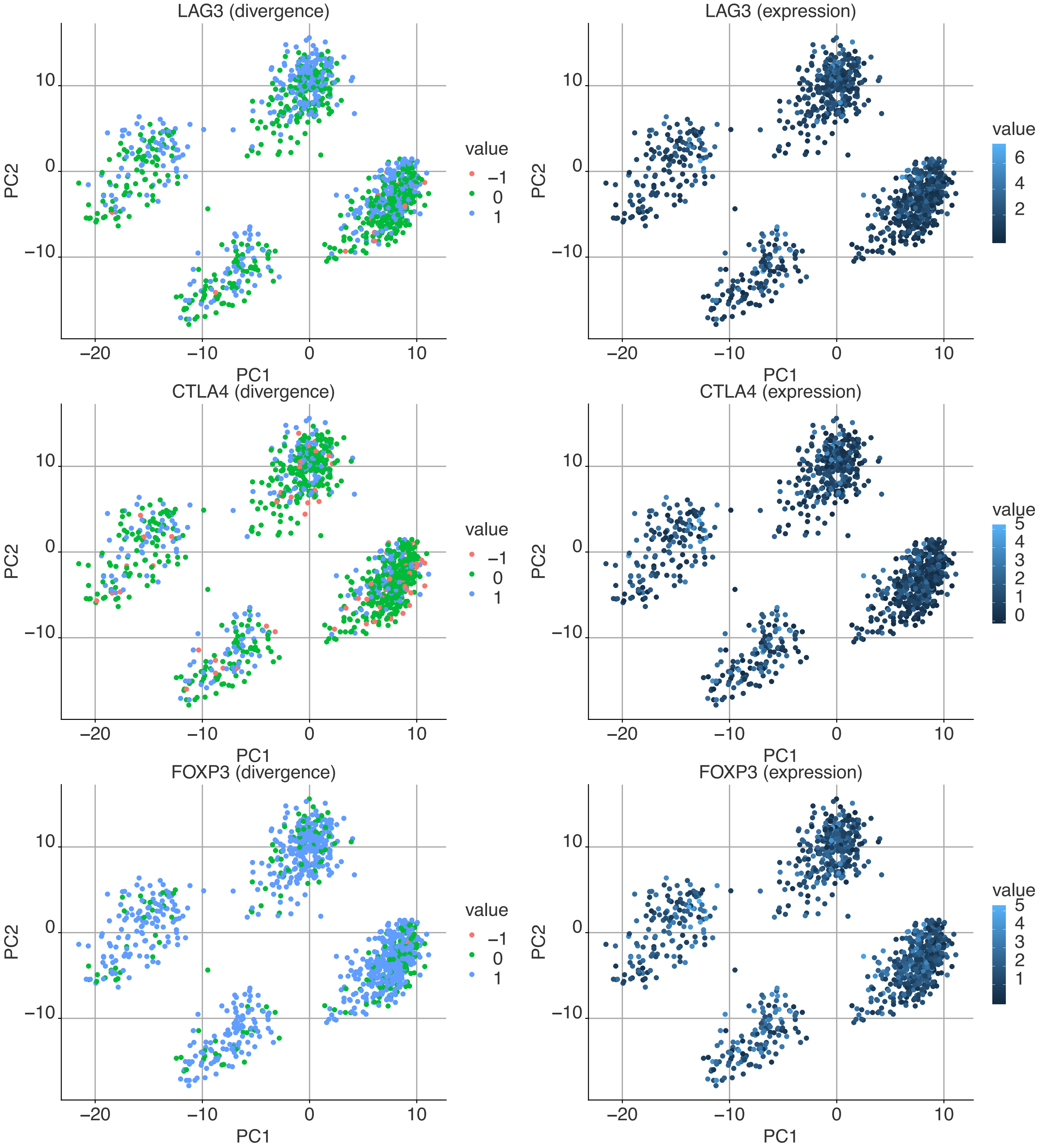

LAG3 (divergence)
LAG3 (expression)
CTLA4 (divergence)
CTLA4 (expression)
FOXP3 (divergence)
FOXP3 (expression)
PC1
PC2
value
−1
0
1
−20
−10
0
10
6
4
2
5
3

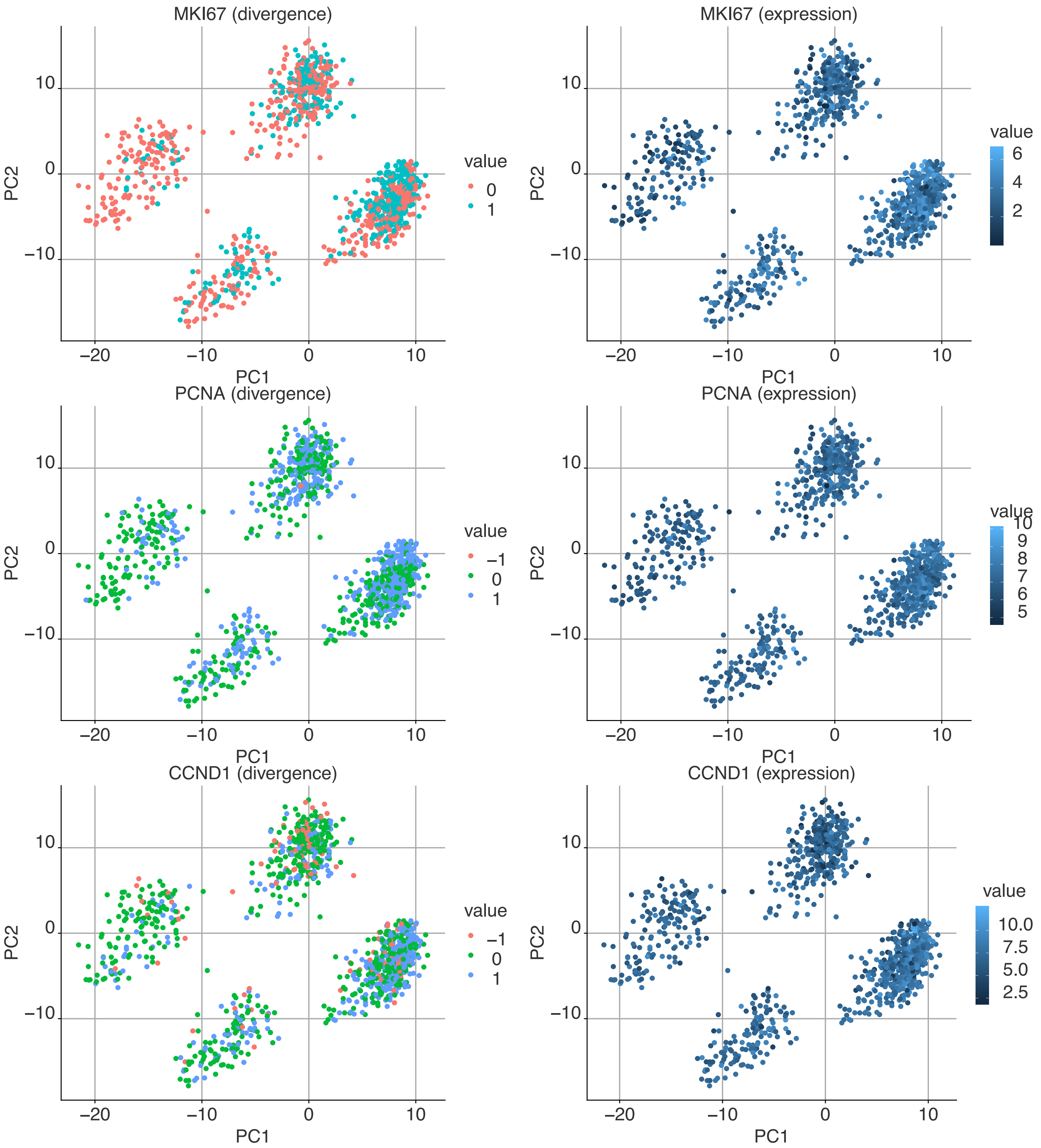
MKI67 (divergence)
MKI67 (expression)
PCNA (divergence)
PCNA (expression)
CCND1 (divergence)
CCND1 (expression)
PC1
PC2
−20
−10
0
10
value
0
1
−1
6
4
2
9
8
7
6
5
10.0
7.5
5.0
2.5

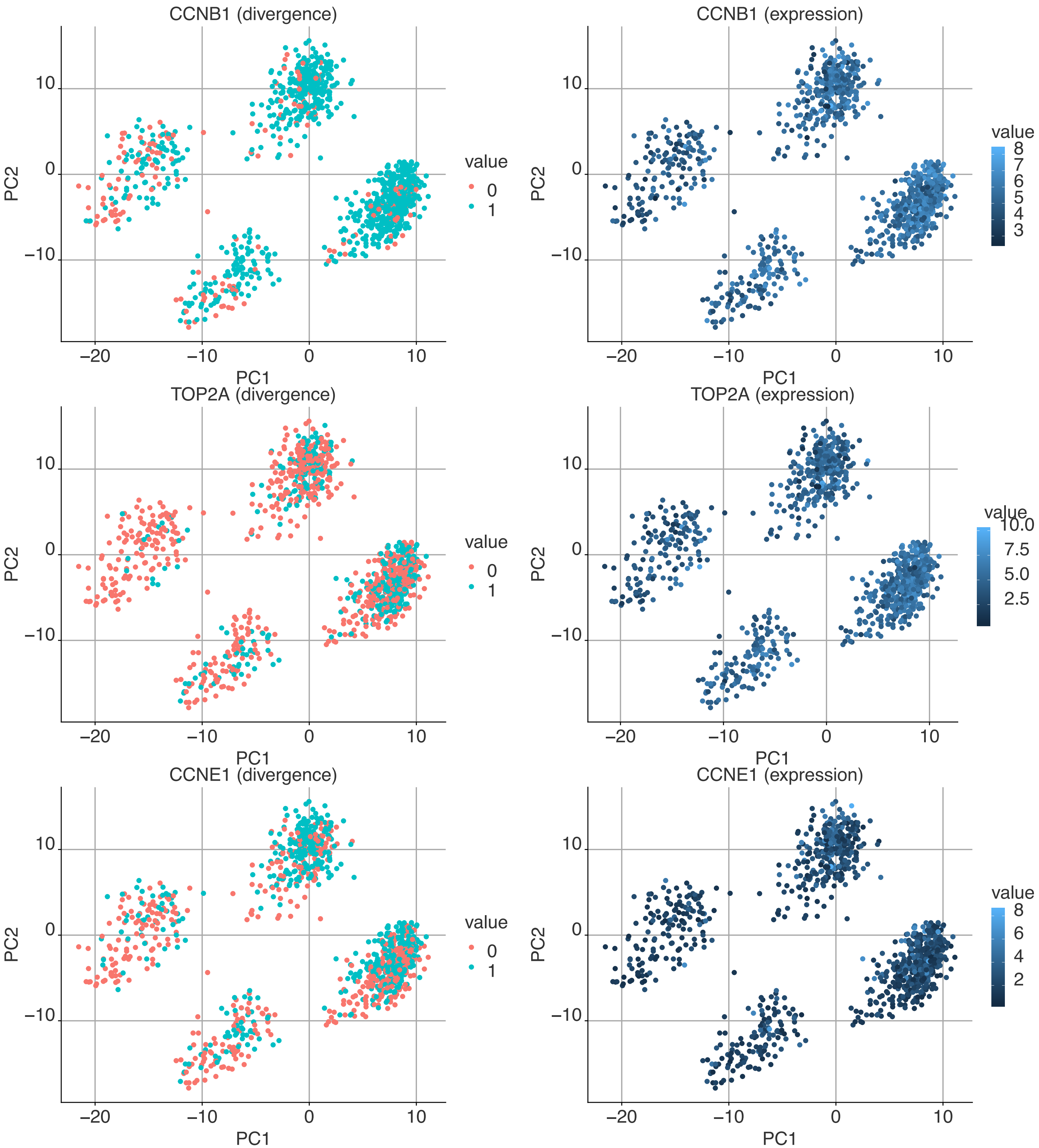
CCNB1 (divergence)
CCNB1 (expression)
TOP2A (divergence)
TOP2A (expression)
CCNE1 (divergence)
CCNE1 (expression)
PC1
PC2
value
0
1
10.0
7.5
5.0
2.5
−20
−10
0
10

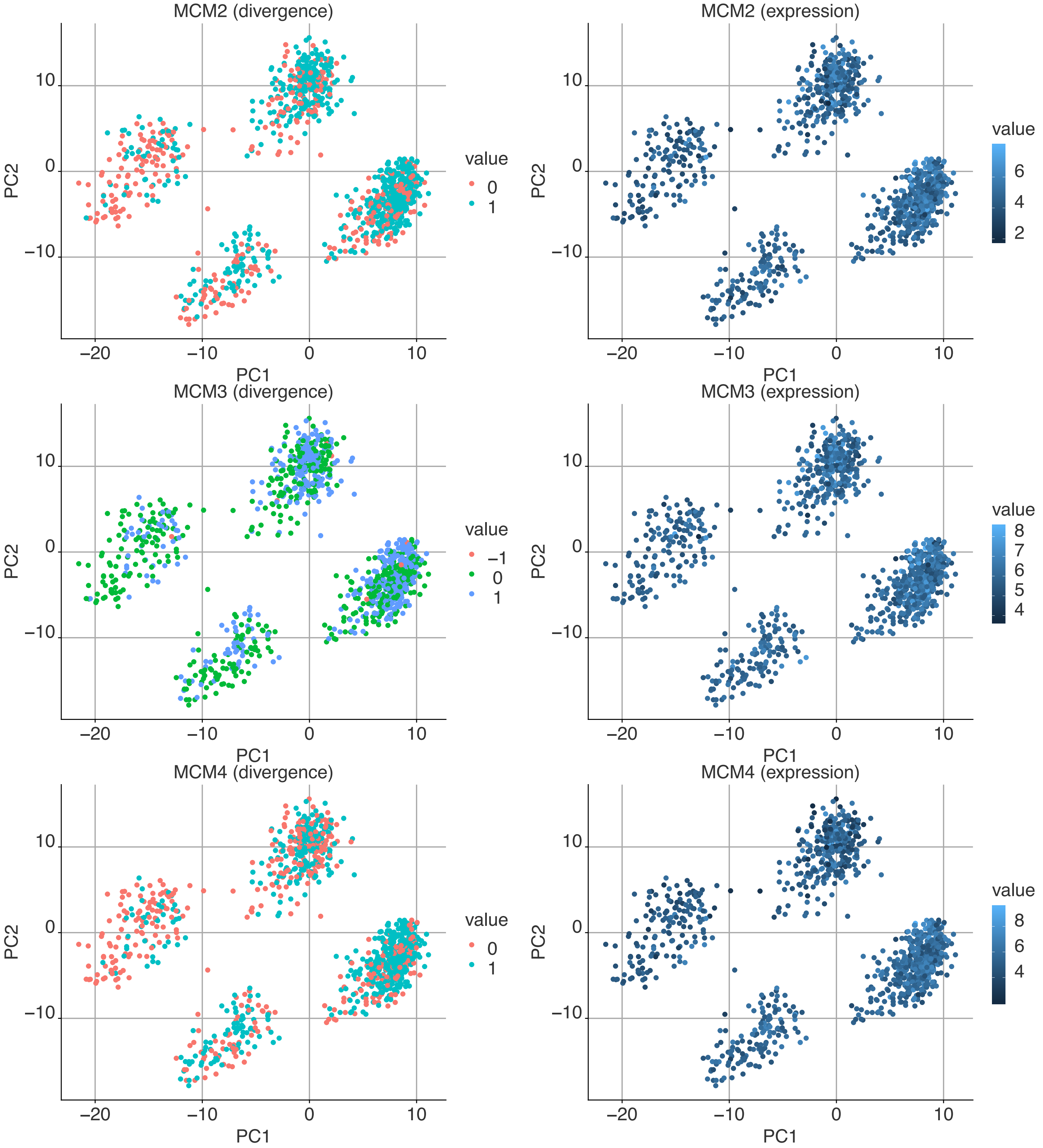

MCM2 (divergence)
MCM2 (expression)
MCM3 (divergence)
MCM3 (expression)
MCM4 (divergence)
MCM4 (expression)
PC1
PC2
value
0
1
−1

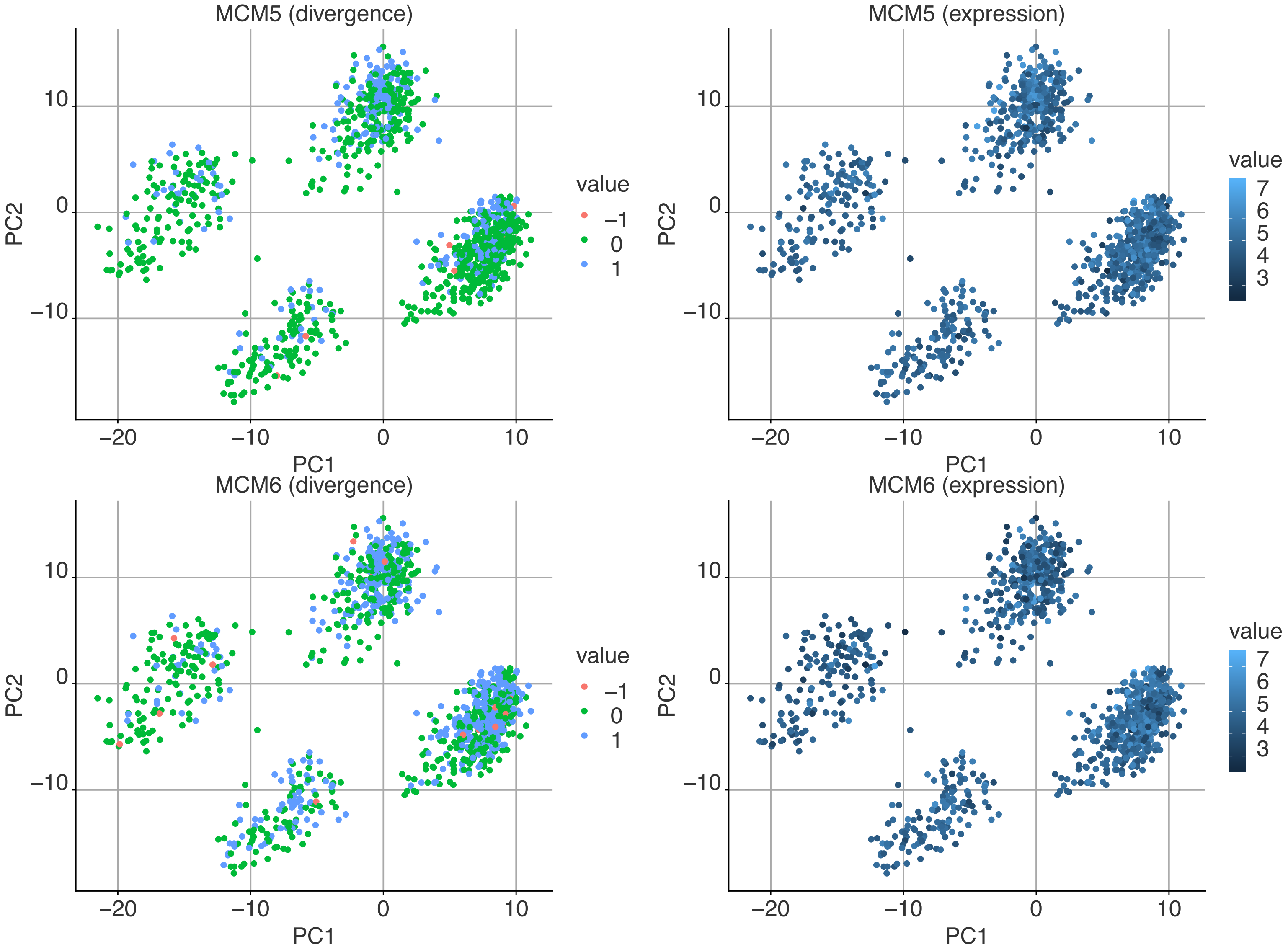

MCM5 (divergence)
MCM5 (expression)
MCM6 (divergence)
MCM6 (expression)
PC1
PC2
−20
−10
0
10
value
−1
0
1
7
6
5
4
3
