# Supplementary figures and images for: Identifying Personalized Metabolic Signatures in Breast Cancer
Source: Metabolites. 2020 Dec 30;11(1):20. doi: 10.3390/metabo11010020 (PMC7823382; doi:10.3390/metabo11010020)

# Cluster 1 vs Normal

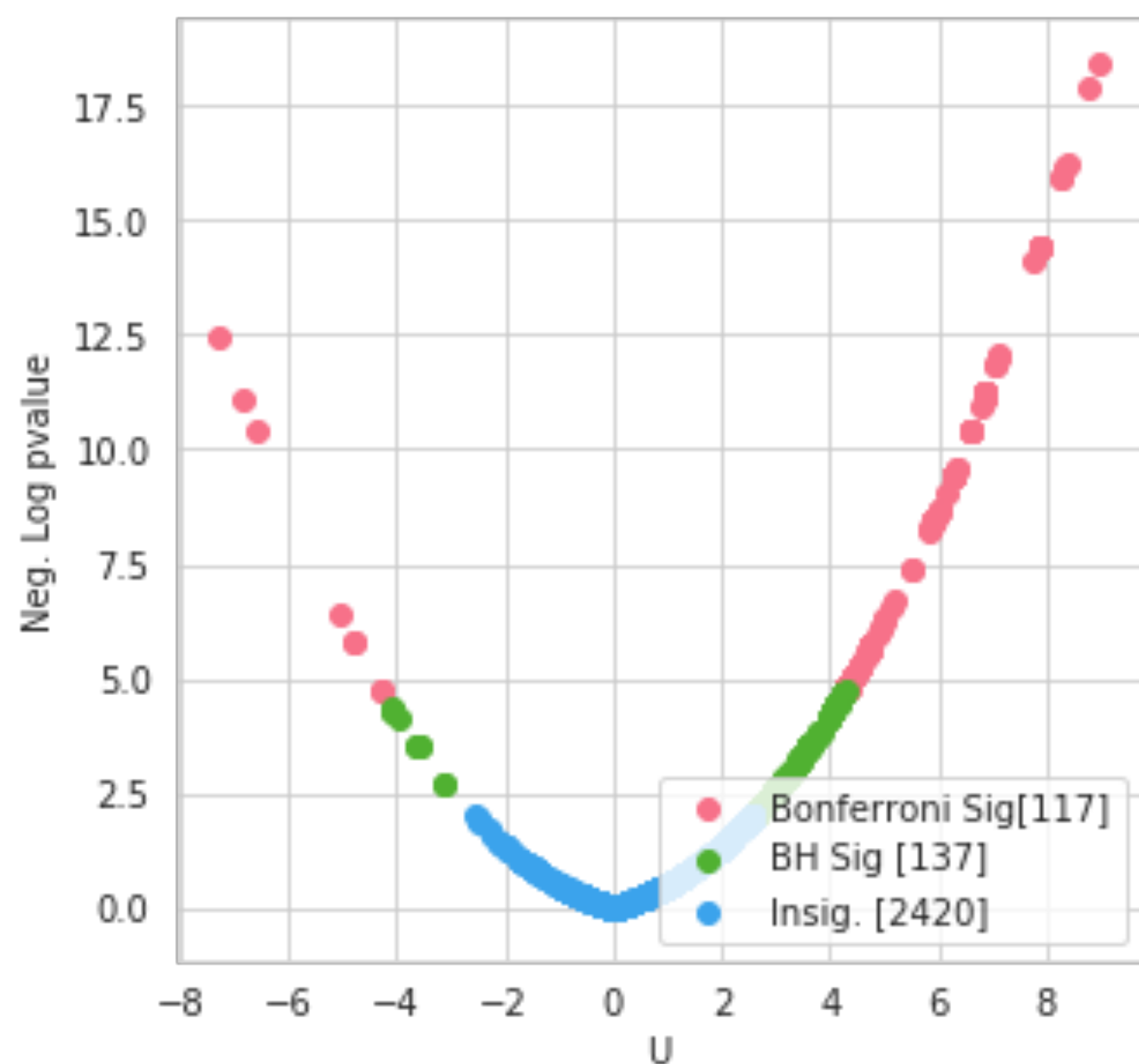

# Cluster 2 vs Normal

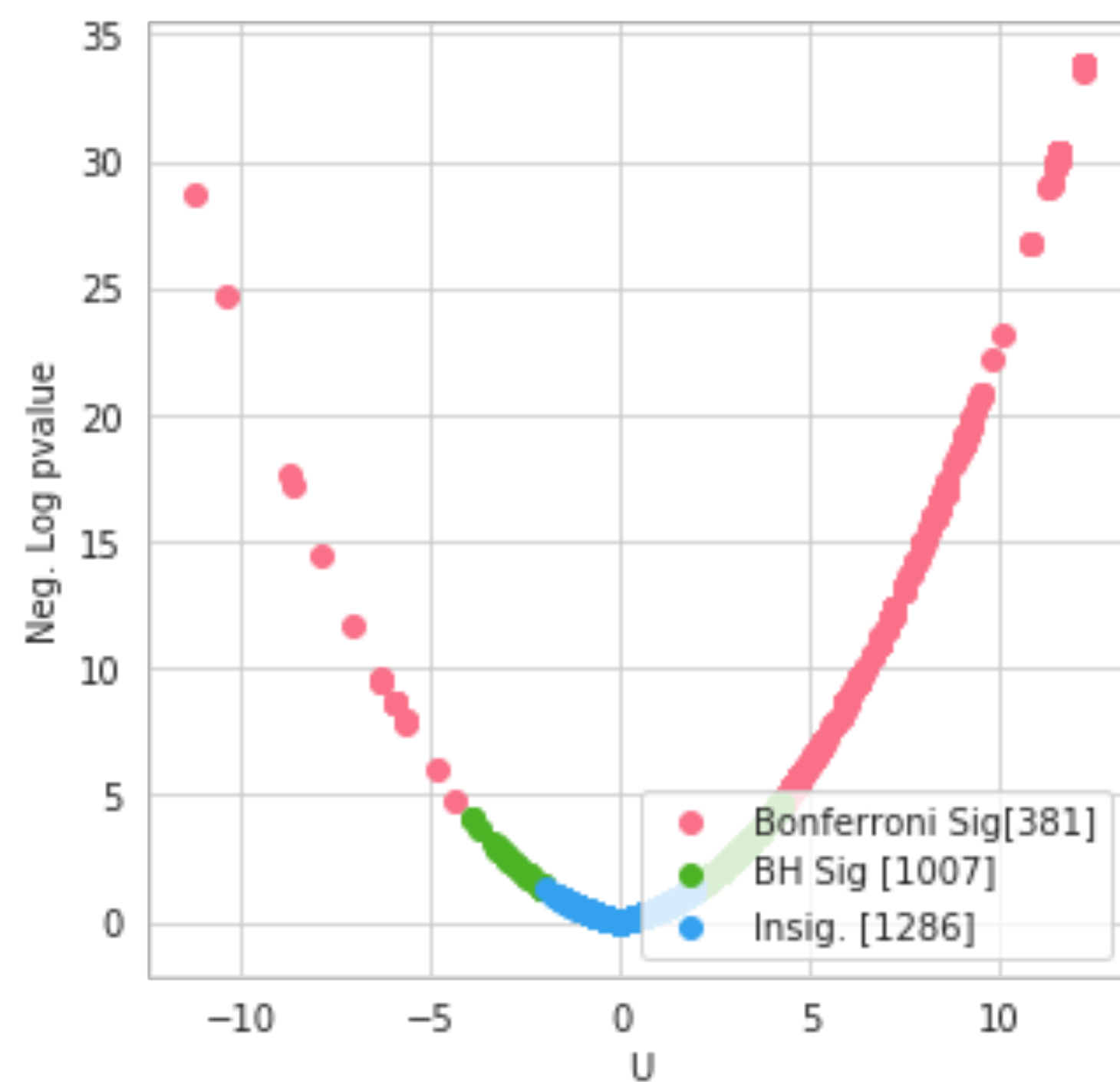

# Cluster 3 vs Normal

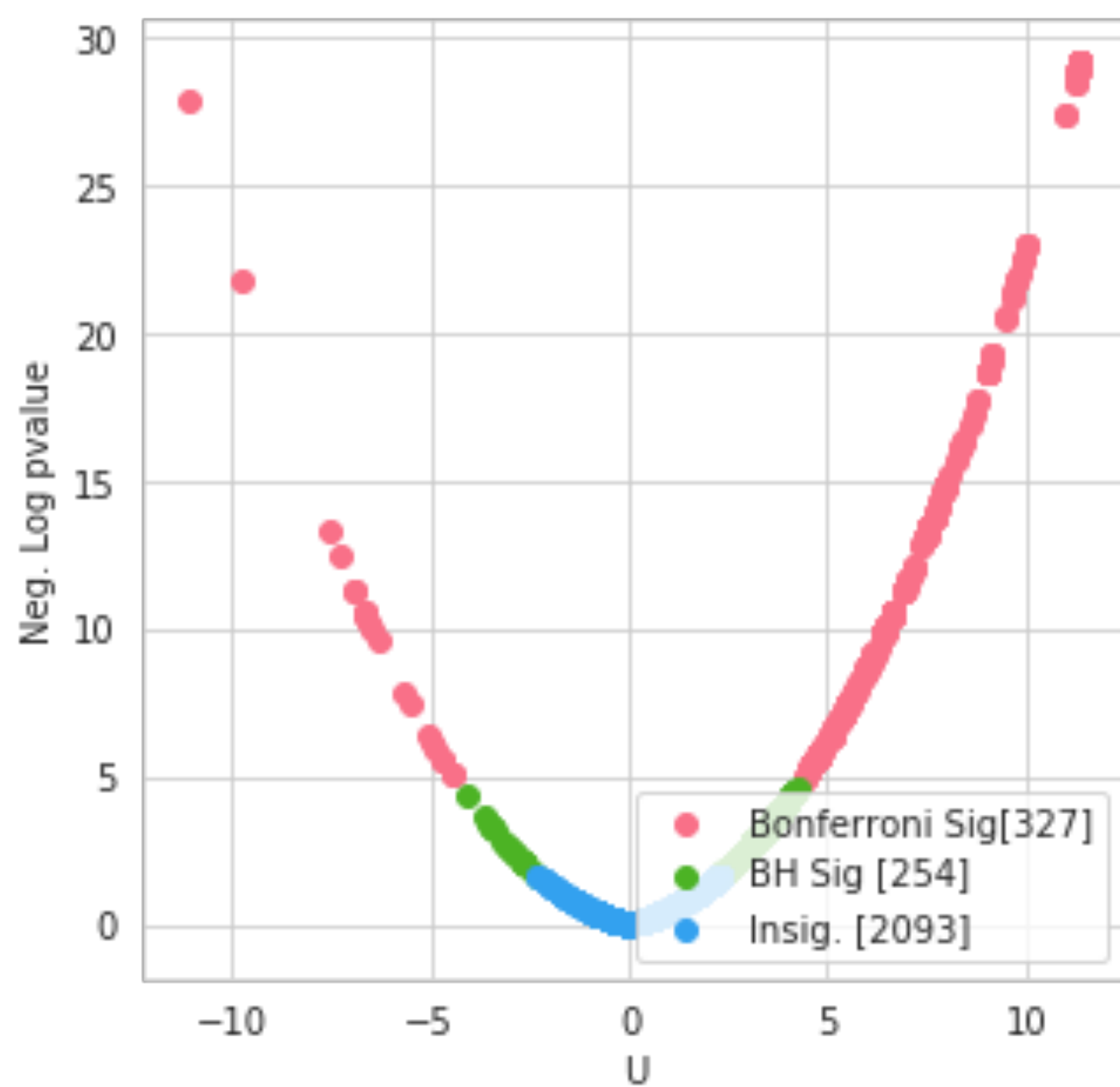

# Cluster 4 vs Normal

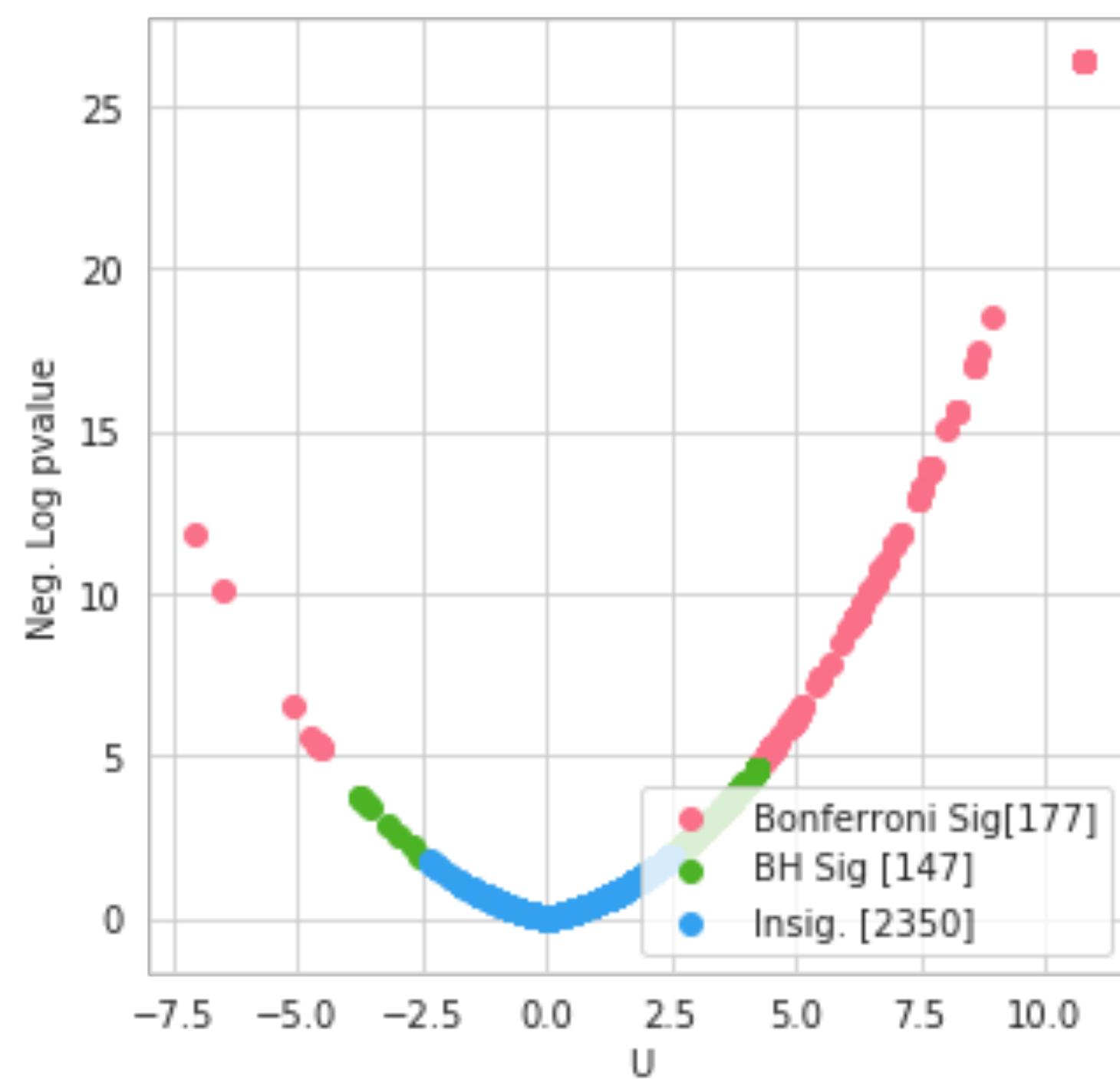

Supplement: Supplementary file 1 [file metabolites-11-00020-s001.zip › Supplementary_files_revision/Supplementary_figure_3.pdf]

Optimal number of clusters

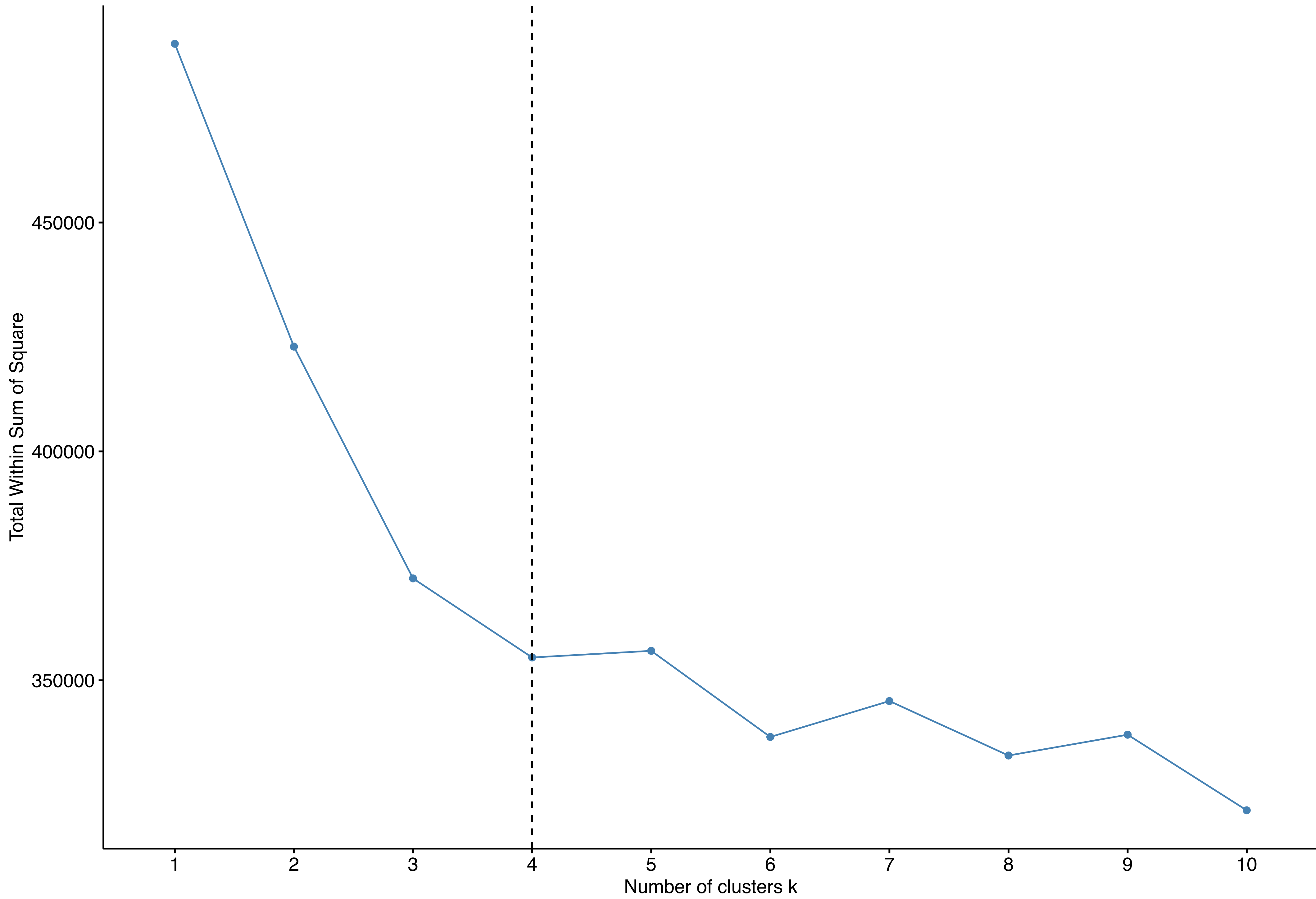

Supplement: Supplementary file 1 [file metabolites-11-00020-s001.zip › Supplementary_files_revision/Supplementary_figure_2.pdf]

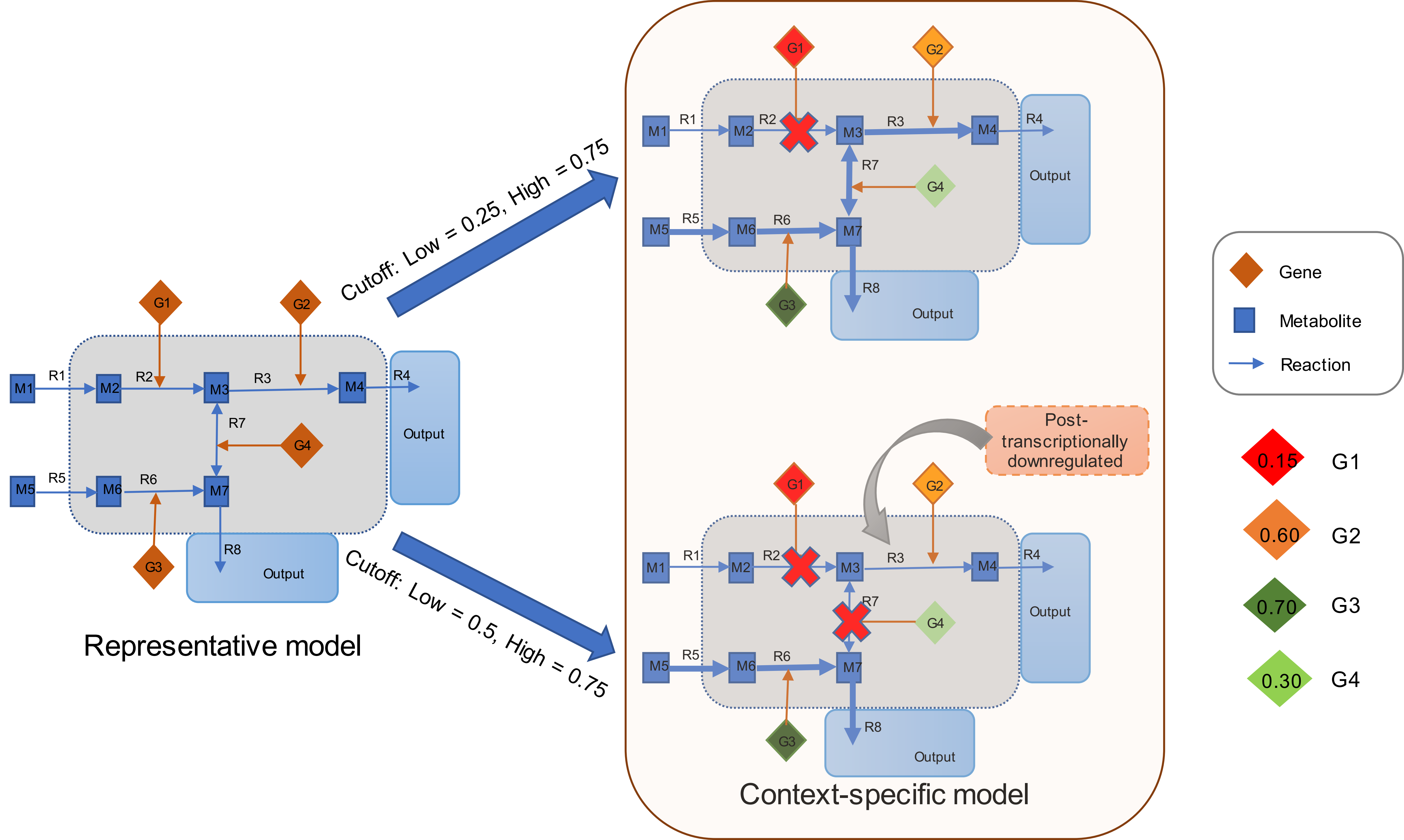

Supplement: Supplementary file 1 [file metabolites-11-00020-s001.zip › Supplementary_files_revision/Supplementary_figure_1.pdf]

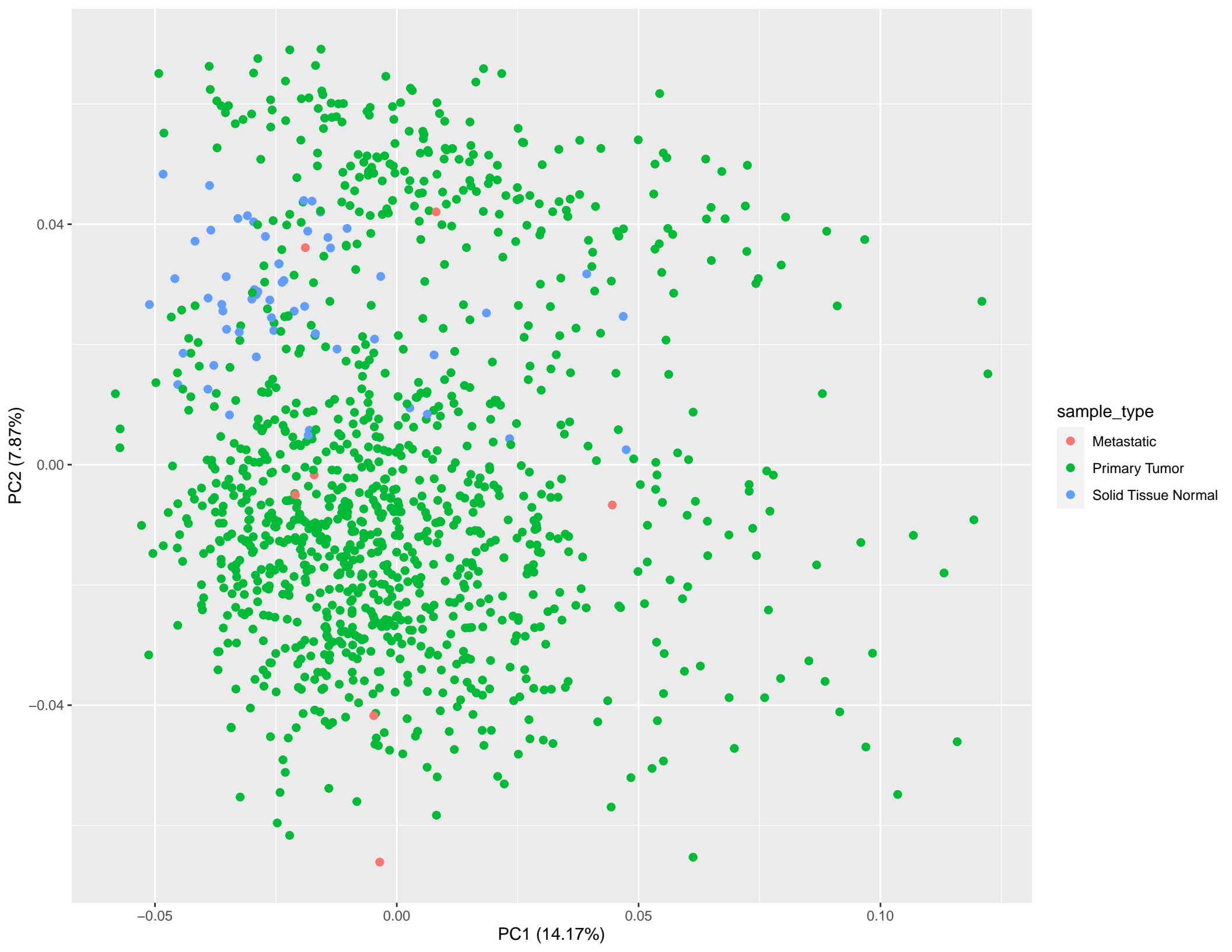

Supplement: Supplementary file 1 [file metabolites-11-00020-s001.zip › Supplementary_files_revision/Supplementary_figure_4.pdf]
